# Supplementary material for: Temporal dynamics of short-term neural adaptation across human visual cortex
Source: PLoS Comput Biol. 2024 May 30;20(5):e1012161. doi: 10.1371/journal.pcbi.1012161 (PMC11166327; doi:10.1371/journal.pcbi.1012161)
Supplement: S1 Fig — A: Cross-validated explained variance (coefficient of determination) across all stimulus conditions for the DN model compared with a two-channel model from [24] (linear + quadratic, L+Q) and [44] (adaptation + sigmoid, A+S) plotted per visual area (V1-V3, VOTC and LOTC). Category-selective scaling is either omitted or included during model fitting. The DN model predicts neural responses to a higher degree compared to both implementations of the two-channel models. Category-dependent scaling further improves model fits. B: Top, Average, normalized broadband iEEG responses (80–200 Hz) for electrodes assigned to V1-V3 (n = 17), VOTC (n = 15) and LOTC (n = 47) to single stimuli (gray). Responses are shown separately for a stimulus duration of 267 and 533 ms. The TTC model predicts an offset response which is not present in the neural data (black arrow). C: Full-width at half maximum, computed for each stimulus duration. The TTC model predicts narrower response widths for VOTC compared to what is observed in the neural data. D: left, Recovery from adaptation computed as the ratio of the Area Under the Curve (AUC) between the first and second response derived from the neural data. The fitted curves express the degree of recovery as a function of the ISI (see Materials and methods, Summary metrics). Right, Same as left for the TTC model. The TTC model poorly aligns with the neural data and predicts an overall higher degree of recovery from RS with area-dependent differences for short as opposed to long ISIs. Data points indicate medians and error bars indicate 68% confidence interval across 1000 samples derived from the bootstrapped timecourses. Panel A can be reproduced by mkSuppFigure1.py. Panel BC and D can be reproduced by mkSuppFigure4.py and mkFigure5_6.py, respectively. (PDF) [file pcbi.1012161.s001.pdf]

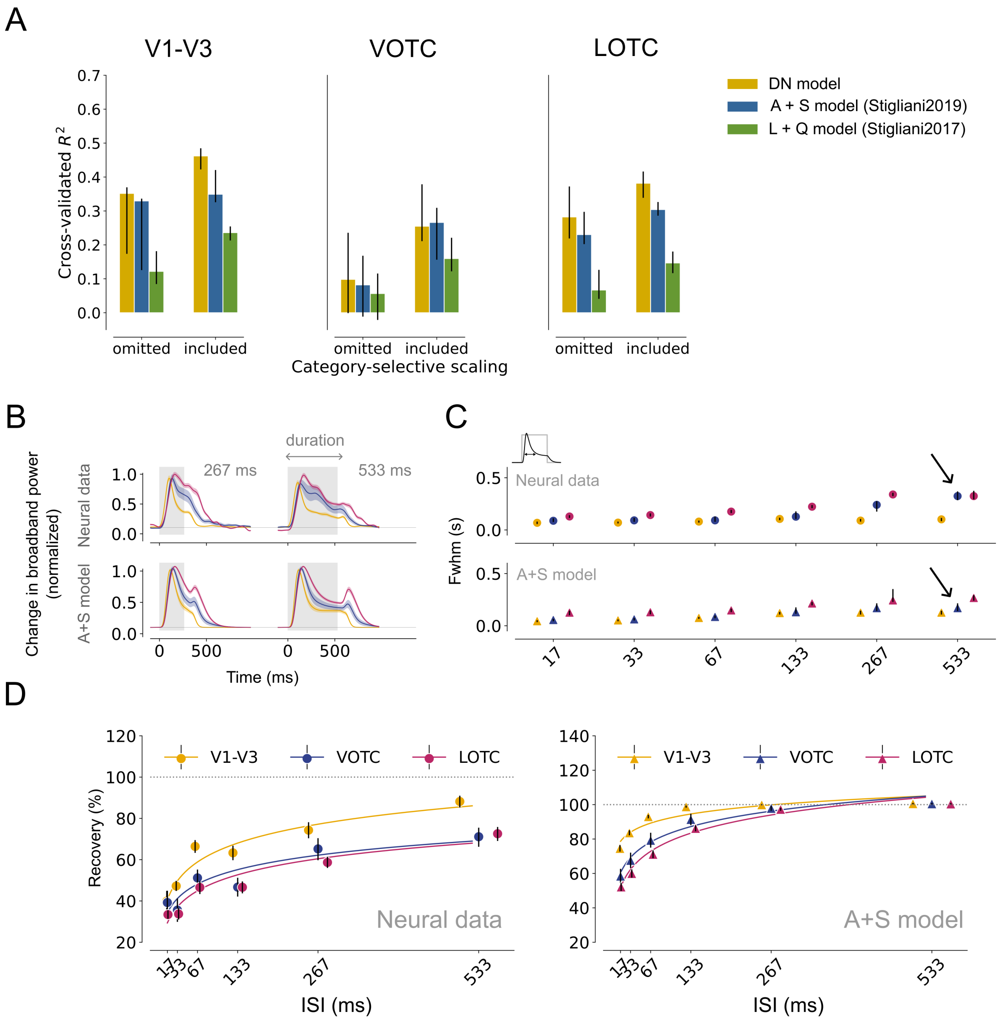

S Fig 1. The two-temporal channel model with adaptation and sigmoidal nonlinearities (A+S, [44]) fails to capture transient-sustained dynamics and repetition suppression (RS) observed in neural responses. A: Cross-validated explained variance (coefficient of determination) across all stimulus conditions for the DN model compared with a two-channel model from [24] (linear + quadratic, L+Q) and [44] (adaptation + sigmoid, A+S) plotted per visual area (V1-V3, VOTC and LOTC). Category-selective scaling is either omitted or included during model fitting. The DN model predicts neural responses to a higher degree compared to both implementations of the two-channel models. Category-dependent scaling further improves model fits. B: Top, Average, normalized broadband iEEG responses (80-200 Hz) for electrodes assigned to V1-V3 (n = 17), VOTC (n = 15) and LOTC (n = 47) to single stimuli (gray). Responses are shown separately for a stimulus duration of 267 and 533 ms. The TTC model predicts an offset response which is not present in the neural data (black arrow). C: Full-width at half maximum, computed for each stimulus duration. The TTC model predicts narrower response widths for VOTC compared to what is observed in the neural data. D: left, Recovery from adaptation computed as the ratio of the Area Under the Curve (AUC) between the first and second response derived from the neural data. The fitted curves express the degree of recovery as a function of the ISI (see Materials and methods, Summary metrics). Right, Same as left for the TTC model. The TTC model poorly aligns with the neural data and predicts an overall higher degree of recovery from RS with area-dependent differences for short as opposed to long ISIs. Data points indicate medians and error bars indicate 68% confidence interval across 1000 samples derived from the bootstrapped timecourses. Panel A can be reproduced by mkSuppFigure1.py. Panel BC and D can be reproduced by mkSuppFigure4.py and mkFigure5_6.py, respectively.
