# Supplementary material for: Temporal dynamics of short-term neural adaptation across human visual cortex
Source: PLoS Comput Biol. 2024 May 30;20(5):e1012161. doi: 10.1371/journal.pcbi.1012161 (PMC11166327; doi:10.1371/journal.pcbi.1012161)
Supplement: S2 Fig — (previous page). A: Top, Average, normalized broadband iEEG responses (80–200 Hz) for electrodes assigned to V1-V3 (n = 17), VOTC (n = 15) and LOTC (n = 47) to single, preferred stimuli (gray). Responses are shown separately per duration from shortest (17ms, left) to longest (533 ms, right). Bottom, DN model predictions for the same conditions. The shapes of the neural time courses differ between visual areas and are accurately captured by the DN model. Time courses were smoothed with a Gaussian kernel with standard deviation of σ = 10; the shaded regions indicate 68% confidence interval across 1000 bootstrapped timecourses (see Materials and methods, Bootstrapping procedure and statistical testing). B-C: Summary metrics plotted per visual area derived from the neural responses (circle marker) or model time courses (triangle marker). Time-to-peak (B) computed to the longest duration (533 ms). Full-width at half maximum (C), computed for each stimulus duration. For higher visual areas, results suggest that neural responses show stronger reduction at stimulus offset for preferred compared to non-preferred stimuli (black arrow in panel A and D), which is captured by the DN model. Data points indicate medians and error bars indicate 68% confidence interval across 1000 samples derived from the bootstrapped timecourses. Bootstrap test, * = p < 0.05 (two-tailed, Bonferroni-corrected). D-F: Same as A-C for trials showing non-preferred stimuli. This figure can be reproduced by mkFigure4.py. (PDF) [file pcbi.1012161.s002.pdf]

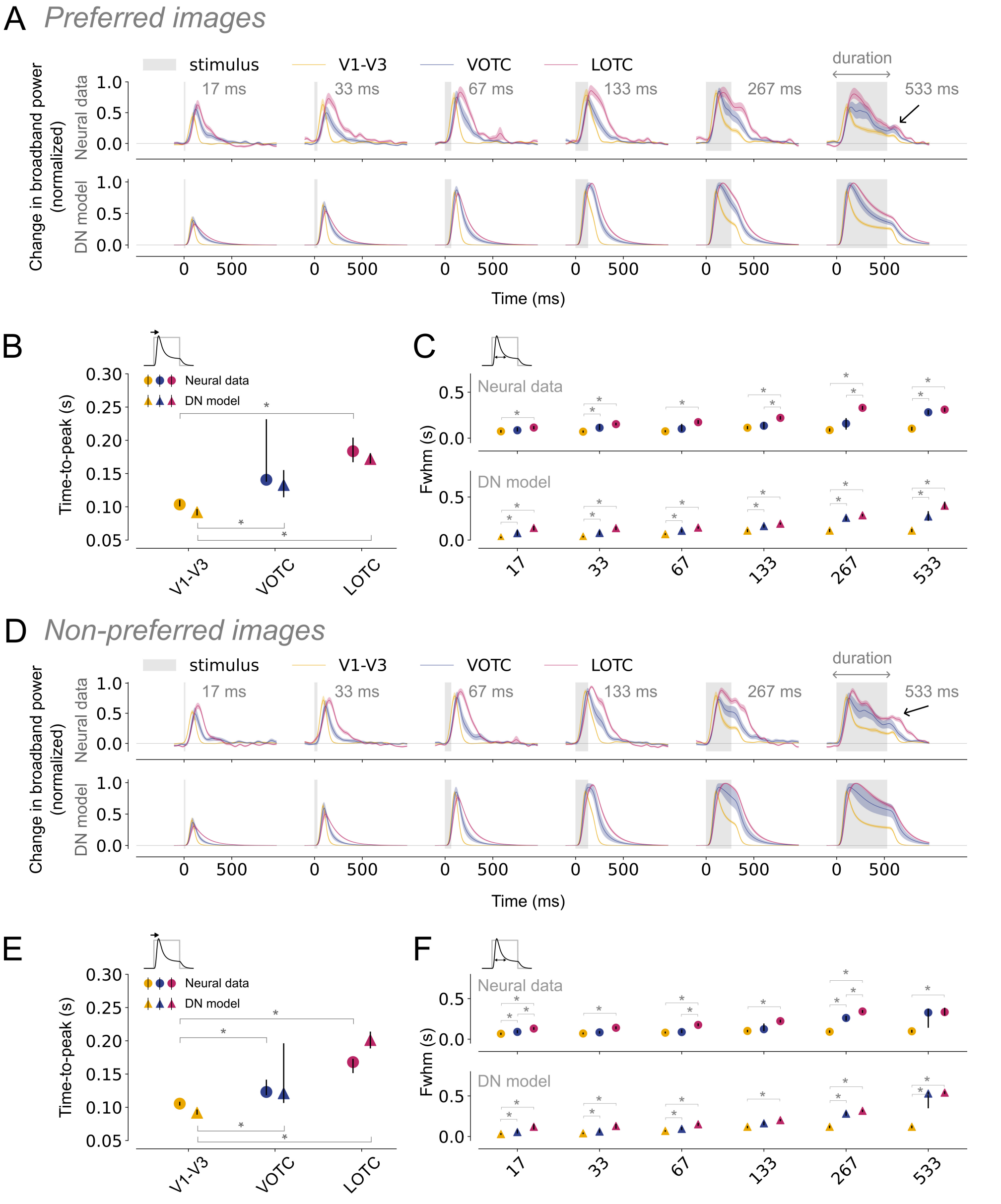

S Fig 2. Slower rise and prolonged responses in higher visual areas for single stimuli for preferred and non-preferred stimuli. (*previous page*). A: Top, Average, normalized broadband iEEG responses (80-200 Hz) for electrodes assigned to V1-V3 (n = 17), VOTC (n = 15) and LOTC (n = 47) to single, preferred stimuli (gray). Responses are shown separately per duration from shortest (17ms, left) to longest (533 ms, right). Bottom, DN model predictions for the same conditions. The shapes of the neural time courses differ between visual areas and are accurately captured by the DN model. Time courses were smoothed with a Gaussian kernel with standard deviation of $\sigma = 10$; the shaded regions indicate 68% confidence interval across 1000 bootstrapped timecourses (see Materials and methods, Bootstrapping procedure and statistical testing). B-C: Summary metrics plotted per visual area derived from the neural responses (circle marker) or model time courses (triangle marker). Time-to-peak (B) computed to the longest duration (533 ms). Full-width at half maximum (C), computed for each stimulus duration. For higher visual areas, results suggest that neural responses show stronger reduction at stimulus offset for preferred compared to non-preferred stimuli (black arrow in panel A and D), which is captured by the DN model. Data points indicate medians and error bars indicate 68% confidence interval across 1000 samples derived from the bootstrapped timecourses. Bootstrap test, $* = p < 0.05$ (two-tailed, Bonferroni-corrected). D-F: Same as A-C for trials showing non-preferred stimuli. This figure can be reproduced by mkFigure4.py.
