# Supplementary material for: Temporal dynamics of short-term neural adaptation across human visual cortex
Source: PLoS Comput Biol. 2024 May 30;20(5):e1012161. doi: 10.1371/journal.pcbi.1012161 (PMC11166327; doi:10.1371/journal.pcbi.1012161)
Supplement: S3 Fig — A. Top, the broadband time course of an example electrode averaged over all repetition trials plotted separately for each ISI. Bottom, an estimate of the response to the first and second stimulus. A robust response to the first stimulus is obtained by averaging the response time courses for the 134 ms duration stimulus and each of the repetition stimuli from trial onset up to the onset of the second stimulus. The estimate of the response to the second stimulus is computed by subtracting the average time course of the first stimulus from the ISI varying stimulus responses. B: The recovery from neural adaptation is computed and defined as the Area Under the Curve (AUC) of the second pulse proportional to AUC of the first pulse, as a function of the ISI. This figure can be reproduced by mkSuppFigure3.py. (PDF) [file pcbi.1012161.s003.pdf]

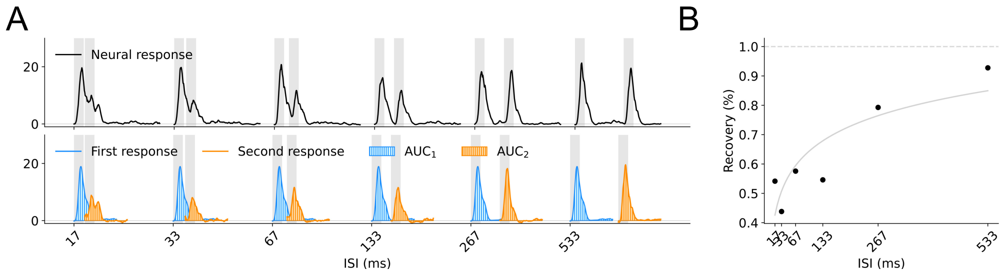

S Fig 3. Extract degree of recovery from repetition suppression. A. Top, the broadband time course of an example electrode averaged over all repetition trials plotted separately for each ISI. Bottom, an estimate of the response to the first and second stimulus. A robust response to the first stimulus is obtained by averaging the response time courses for the 134 ms duration stimulus and each of the repetition stimuli from trial onset up to the onset of the second stimulus. The estimate of the response to the second stimulus is computed by subtracting the average time course of the first stimulus from the ISI varying stimulus responses. B: The recovery from neural adaptation is computed and defined as the Area Under the Curve (AUC) of the second pulse proportional to AUC of the first pulse, as a function of the ISI. This figure can be reproduced by mkSuppFigure3.py.
