# Supplementary material for: Temporal dynamics of short-term neural adaptation across human visual cortex
Source: PLoS Comput Biol. 2024 May 30;20(5):e1012161. doi: 10.1371/journal.pcbi.1012161 (PMC11166327; doi:10.1371/journal.pcbi.1012161)
Supplement: S5 Fig — A. Estimated, normalized response to the second stimulus for V1-V3, VOTC and LOTC. For each visual area, the left panel shows the neural data and the right panel shows the model prediction. Time courses were obtained using a bootstrapping procedure (n = 1000, see Materials and methods, Bootstrapping procedure and statistical testing). Recovery from adaptation gradually increases as the ISI becomes longer, and the rate of recovery is higher for V1-V3 compared to VOTC and LOTC in both the neural data and the DN model. B: left, Recovery from adaptation for V1-V3 (n = 17), VOTC (n = 15) and LOTC (n = 47), computed as the ratio of the Area Under the Curve (AUC) between the first and second response. The fitted curves express the degree of recovery as a function of the ISI (see Materials and methods, Summary metrics). Higher visual areas show stronger RS and slower recovery from adaptation. Area-related differences are less pronounces compared to preferred stimulus trials (S5 Fig). Right, model predictions for the same data. The model is able to capture area-specific recovery from adaptation. B-C: Summary metrics plotted per visual area derived from the neural responses (circle marker) or model time courses (triangle marker). Average recovery (B) from adaptation for each area, computed by averaging the AUC ratios between the first and second stimulus over all ISIs. The long-term recovery (C) reflects the amount of recovery for an ISI of 1s, obtained by extrapolating the fitted line. Higher visual areas show stronger RS and a slower recovery rate which is accurately predicted by the DN model. Data points indicate medians and error bars indicate 68% confidence interval across 1000 samples derived from the bootstrapped timecourses. Bootstrap test, * = p < 0.05 (two-tailed, Bonferroni-corrected). This figure can be reproduced by mkFigure5_6.py. (PDF) [file pcbi.1012161.s005.pdf]

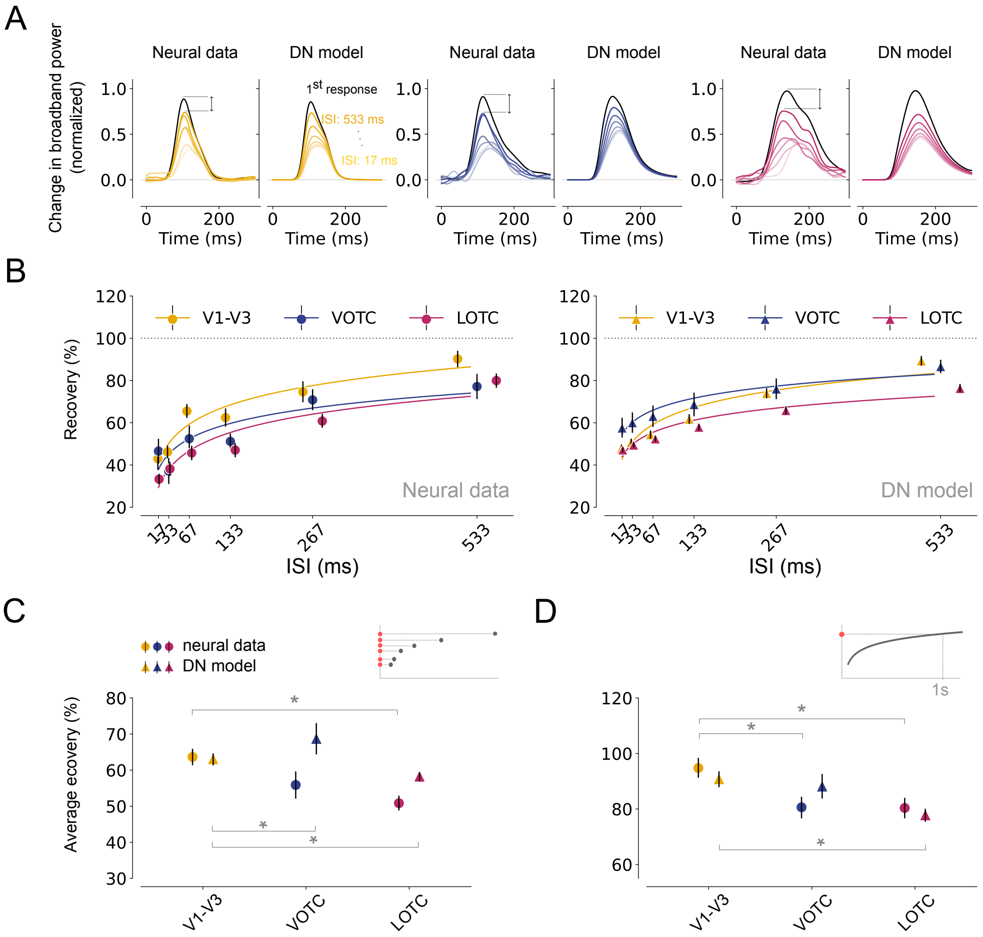

S Fig 5. Differences in recovery from repetition suppression across visual areas for non-preferred stimuli. A. Estimated, normalized response to the second stimulus for V1-V3, VOTC and LOTC. For each visual area, the left panel shows the neural data and the right panel shows the model prediction. Time courses were obtained using a bootstrapping procedure ($n = 1000$, see Materials and methods, Bootstrapping procedure and statistical testing). Recovery from adaptation gradually increases as the ISI becomes longer, and the rate of recovery is higher for V1-V3 compared to VOTC and LOTC in both the neural data and the DN model. B: left, Recovery from adaptation for V1-V3 (n = 17), VOTC (n = 15) and LOTC (n = 47), computed as the ratio of the Area Under the Curve (AUC) between the first and second response. The fitted curves express the degree of recovery as a function of the ISI (see Materials and methods, Summary metrics). Higher visual areas show stronger RS and slower recovery from adaptation. Area-related differences are less pronounces compared to preferred stimulus trials (**??**). Right, model predictions for the same data. The model is able to capture area-specific recovery from adaptation. B-C: Summary metrics plotted per visual area derived from the neural responses (circle marker) or model time courses (triangle marker). Average recovery (B) from adaptation for each area, computed by averaging the AUC ratios between the first and second stimulus over all ISIs. The long-term recovery (C) reflects the amount of recovery for an ISI of 1s, obtained by extrapolating the fitted line. Higher visual areas show stronger RS and a slower recovery rate which is accurately predicted by the DN model. Data points indicate medians and error bars indicate 68% confidence interval across 1000 samples derived from the bootstrapped timecourses. Bootstrap test, $* = p < 0.05$ (two-tailed, Bonferroni-corrected). This figure can be reproduced by mkFigure5_6.py.
