# Supplementary material for: Temporal dynamics of short-term neural adaptation across human visual cortex
Source: PLoS Comput Biol. 2024 May 30;20(5):e1012161. doi: 10.1371/journal.pcbi.1012161 (PMC11166327; doi:10.1371/journal.pcbi.1012161)
Supplement: S6 Fig — A. Top, Average, broadband responses of category-selective electrodes (threshold d′ = 0.75, n = 12) of trials during which preferred (blue) or non-preferred (red) stimuli were presented in repetition (gray). Time courses were obtained using a bootstrapping procedure (n = 1000, see Materials and methods, Bootstrapping procedure and statistical testing). Responses are shown separately per ISI from shortest (17 ms, left) to longest (533 ms, right). Bottom, DN model predictions for the same data. Time courses differ for preferred and non-preferred stimuli which is captured by the DN model. This figure can be reproduced by mkFigure8.py. (PDF) [file pcbi.1012161.s006.pdf]

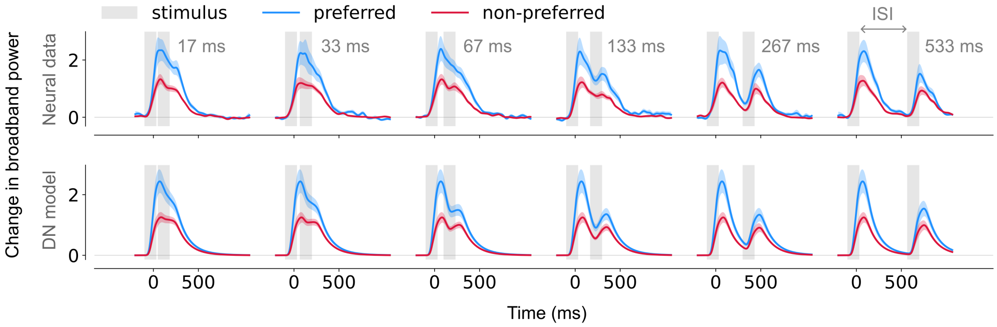

S Fig 6. Differences in recovery from adaptation across stimuli in category-selective areas. A. Top, Average, broadband responses of category-selective electrodes (threshold d' = 0.75, n = 12) of trials during which preferred (blue) or non-preferred (red) stimuli were presented in repetition (gray). Time courses were obtained using a bootstrapping procedure ($n = 1000$, see Materials and methods, Bootstrapping procedure and statistical testing). Responses are shown separately per ISI from shortest (17 ms, left) to longest (533 ms, right). Bottom, DN model predictions for the same data. Time courses differ for preferred and non-preferred stimuli which is captured by the DN model. This figure can be reproduced by mkFigure8.py.
