# Supplementary material for: Temporal dynamics of short-term neural adaptation across human visual cortex
Source: PLoS Comput Biol. 2024 May 30;20(5):e1012161. doi: 10.1371/journal.pcbi.1012161 (PMC11166327; doi:10.1371/journal.pcbi.1012161)
Supplement: S7 Fig — A. Top, Average, normalized broadband responses of category-selective electrodes (threshold d′ = 0.75, n = 12) of trials during which preferred (blue) or non-preferred (red) stimuli were presented in repetition (gray). Time courses were obtained using a bootstrapping procedure (n = 1000, see Materials and methods, Bootstrapping procedure and statistical testing). Responses are shown separately per ISI from shortest (17 ms, left) to longest (533 ms, right). Bottom, DN model predictions for the same data. Time courses differ for preferred and non-preferred stimuli which is captured by the DN model. B. Estimated, normalized response to the second stimulus for trials containing preferred and non-preferred stimuli. Per visual area, the left panel shows the neural data and the right panel shows the model prediction. The rate of recovery is higher for non-preferred compared to preferred stimuli. C: Recovery from adaptation computed as the ratio of the AUC between the first and second response derived from the neural data (left) or DN model predictions (right). The fitted curves express the degree of recovery as a function of the ISI (see Materials and methods, Summary metrics). Responses derived from trials containing preferred stimuli show a stronger degree of RS and the DN model is able to capture stimulus-specific recovery from adaptation. D: Long-term recovery from adaptation derived from the neural responses (circle marker) or DN model (triangle marker), reflecting the amount of recovery for an ISI of 1s. Responses for trials presenting preferred stimuli show stronger RS and a slower recovery rate. Data points indicate medians and error bars indicate 68% confidence interval across 1000 samples derived from the bootstrapped timecourses. Bootstrap test, * = p < 0.05 (two-tailed). This figure can be reproduced by mkFigure8.py. (PDF) [file pcbi.1012161.s007.pdf]

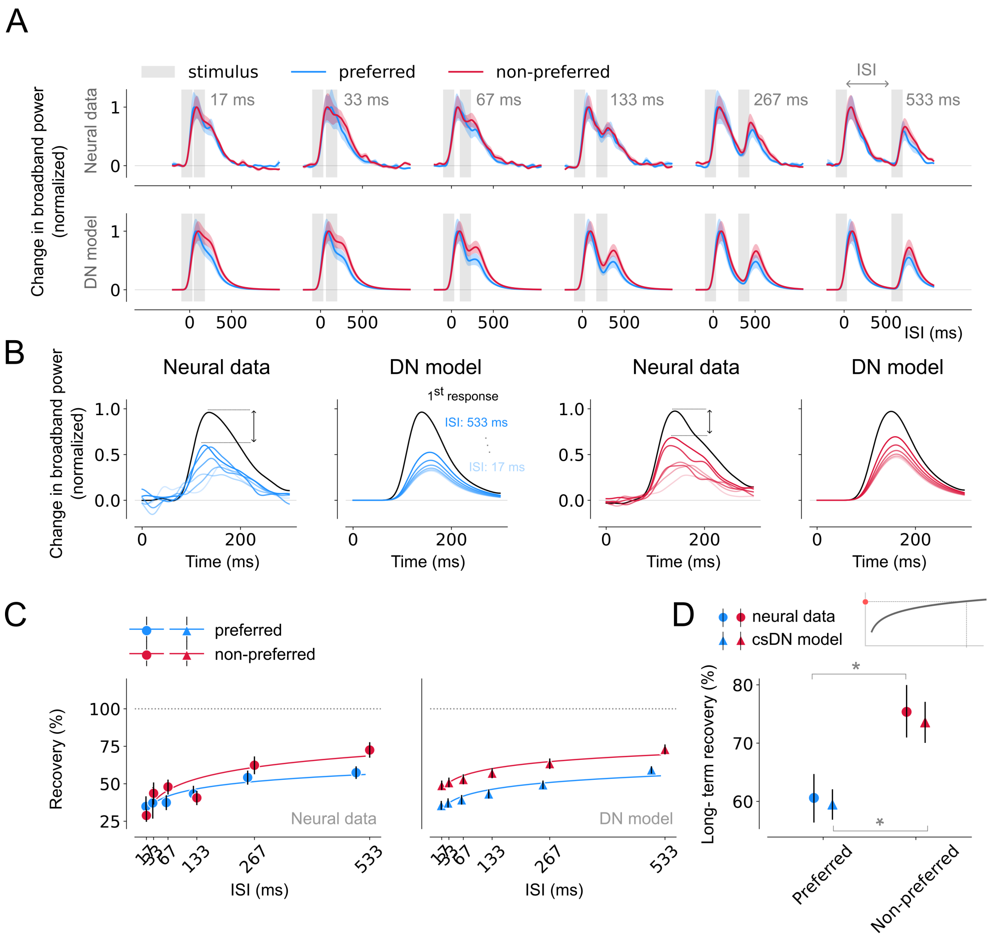

S Fig 7. Differences in recovery from adaptation across stimuli in category-selective areas. A. Top, Average, normalized broadband responses of category-selective electrodes (threshold d' = 0.75, n = 12) of trials during which preferred (blue) or non-preferred (red) stimuli were presented in repetition (gray). Time courses were obtained using a bootstrapping procedure ($n = 1000$, see Materials and methods, Bootstrapping procedure and statistical testing). Responses are shown separately per ISI from shortest (17 ms, left) to longest (533 ms, right). Bottom, DN model predictions for the same data. Time courses differ for preferred and non-preferred stimuli which is captured by the DN model. B. Estimated, normalized response to the second stimulus for trials containing preferred and non-preferred stimuli. Per visual area, the left panel shows the neural data and the right panel shows the model prediction. The rate of recovery is higher for non-preferred compared to preferred stimuli. C: Recovery from adaptation computed as the ratio of the AUC between the first and second response derived from the neural data (left) or DN model predictions (right). The fitted curves express the degree of recovery as a function of the ISI (see Materials and methods, Summary metrics). Responses derived from trials containing preferred stimuli show a stronger degree of RS and the DN model is able to capture stimulus-specific recovery from adaptation. D: Long-term recovery from adaptation derived from the neural responses (circle marker) or DN model (triangle marker), reflecting the amount of recovery for an ISI of 1s. Responses for trials presenting preferred stimuli show stronger RS and a slower recovery rate. Data points indicate medians and error bars indicate 68% confidence interval across 1000 samples derived from the bootstrapped timecourses. Bootstrap test, $* = p < 0.05$ (two-tailed). This figure can be reproduced by mkFigure8.py.
