# Supplementary material for: Temporal dynamics of short-term neural adaptation across human visual cortex
Source: PLoS Comput Biol. 2024 May 30;20(5):e1012161. doi: 10.1371/journal.pcbi.1012161 (PMC11166327; doi:10.1371/journal.pcbi.1012161)
Supplement: S9 Fig — (previous page). Electrode positions for subject p11 (A), subject p12 (B), subject p13 (C) and subject p14 (D) overlaid on a pial surface reconstruction with colour-coded predicted visual locations. A surface node in the pial mesh was assigned a colour if it had a non-zero probability of being in a visual region according to a max probability map from [42]. If the electrode had a nonzero probability of being in multiple regions, the region with the highest probability was assigned. For visualization purposes, some retinotopic maps have been merged (e.g., dorsal and ventral parts of V1). The brain surfaces were created using Freesurfer [39] and scripts can be found at https://github.com/WinawerLab/ECoG_utils. L = lateral, M = medial, D = dorsal, V = ventral, A = anterior, P = posterior. (PDF) [file pcbi.1012161.s009.pdf]

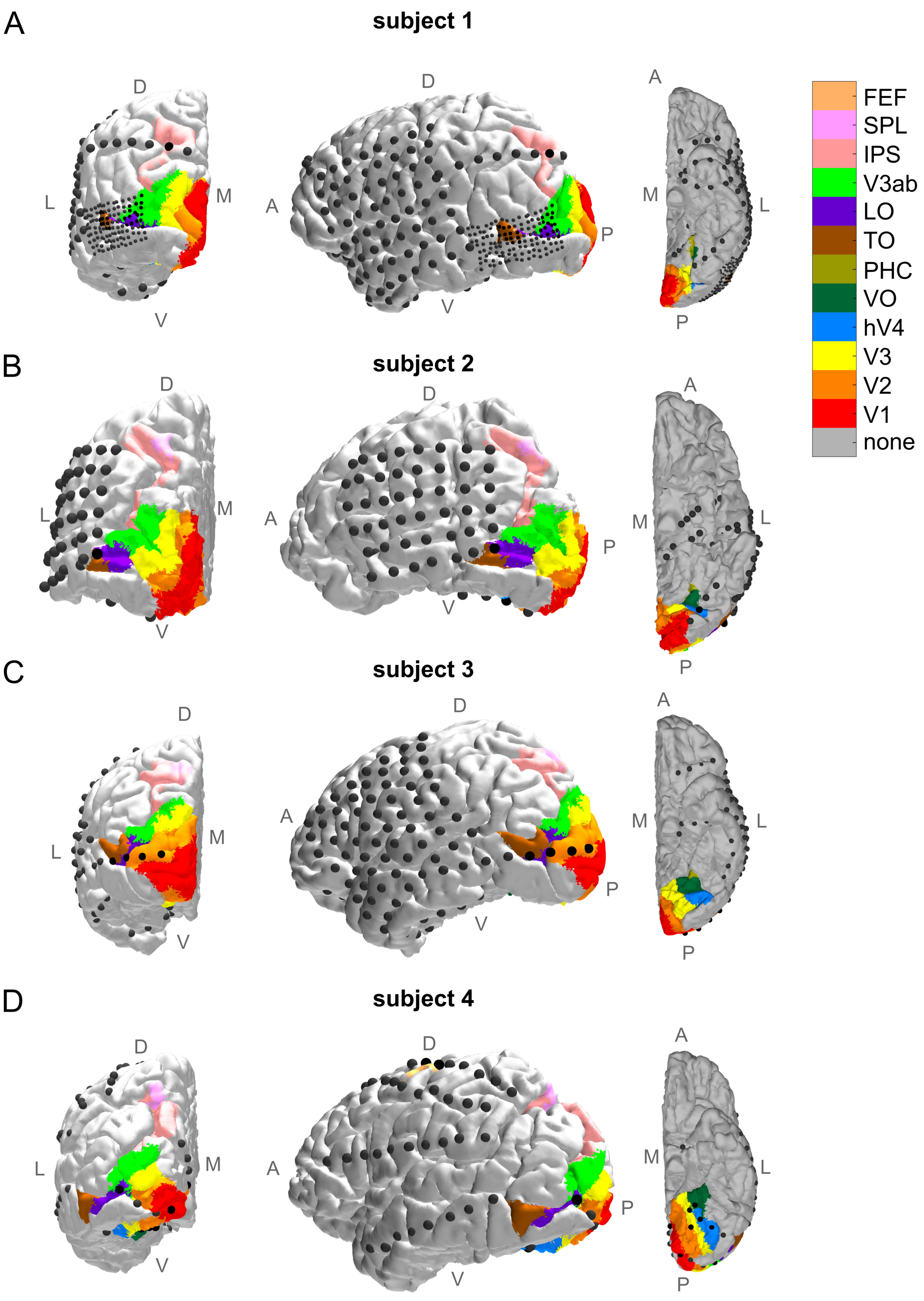

S Fig 9. Electrode positions for individual participants, overlaid on a retinotopic atlas. (*previous page*). Electrode positions for subject p11 (A), subject p12 (B), subject p13 (C) and subject p14 (D) overlaid on a pial surface reconstruction with colour-coded predicted visual locations. A surface node in the pial mesh was assigned a colour if it had a non-zero probability of being in a visual region according to a max probability map from [42]. If the electrode had a nonzero probability of being in multiple regions, the region with the highest probability was assigned. For visualization purposes, some retinotopic maps have been merged (e.g., dorsal and ventral parts of V1). The brain surfaces were created using Freesurfer [39] and scripts can be found at https://github.com/WinawerLab/ECoG_utils. L = lateral, M = medial, D = dorsal, V = ventral, A = anterior, P = posterior.
