# Supplementary material for: Temporal dynamics of short-term neural adaptation across human visual cortex
Source: PLoS Comput Biol. 2024 May 30;20(5):e1012161. doi: 10.1371/journal.pcbi.1012161 (PMC11166327; doi:10.1371/journal.pcbi.1012161)
Supplement: S10 Fig — A. Electrodes with robust visual responses were assigned to early (V1-V3, n = 17), VOTC (n = 15) or LOTC (n = 47) retinotopic areas. Electrodes that were not included in the dataset are shown in black. Electrodes were considered category-selective if the average response for a given image category was higher compared to the other image categories (d′ > 0.75, see Eq 1, Materials and methods, n = 12). B. Same as A for a threshold of d′ for category-selectivity of 1.0 (n = 6). The brain surfaces were created using MNE-Python and can be reproduced by mkFigure2.py. L = lateral, M = medial, D = dorsal, V = ventral, A = anterior, P = posterior. (PDF) [file pcbi.1012161.s010.pdf]

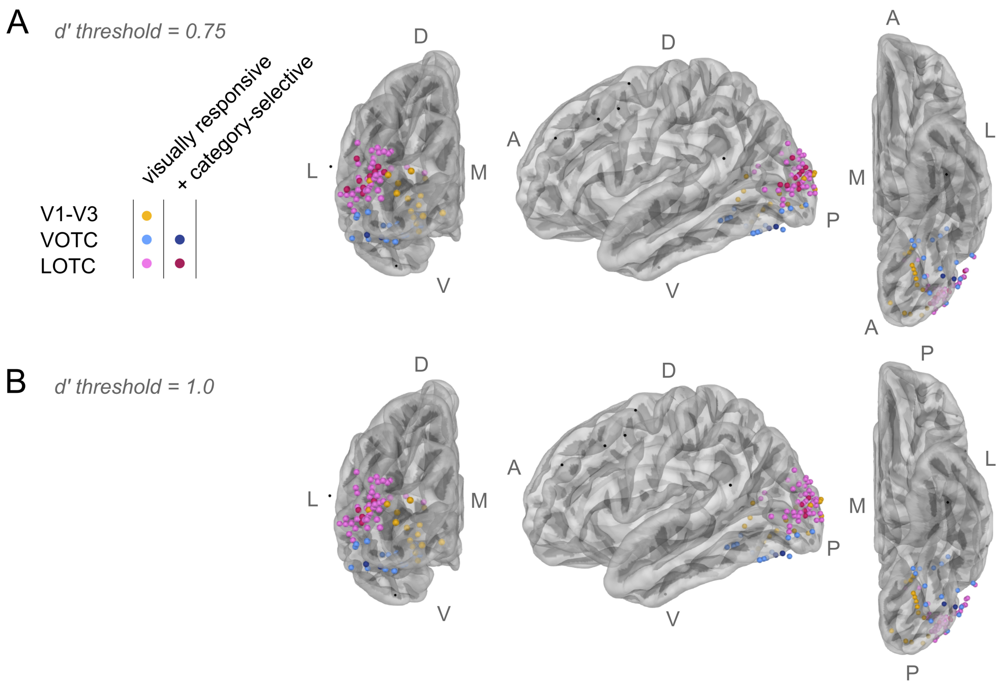

S Fig 10. Electrode positions. A. Electrodes with robust visual responses were assigned to early (V1-V3, n = 17), VOTC (n = 15) or LOTC (n = 47) retinotopic areas. Electrodes that were not included in the dataset are shown in black. Electrodes were considered category-selective if the average response for a given image category was higher compared to the other image categories ($d' > 0.75$, see Eq **??**, Materials and methods, n = 12). B. Same as A for a threshold of d' for category-selectivity of 1.0 (n = 6). The brain surfaces were created using MNE-Python and can be reproduced by mkFigure2.py. L = lateral, M = medial, D = dorsal, V = ventral, A = anterior, P = posterior.
