# Supplementary material for: Temporal dynamics of short-term neural adaptation across human visual cortex
Source: PLoS Comput Biol. 2024 May 30;20(5):e1012161. doi: 10.1371/journal.pcbi.1012161 (PMC11166327; doi:10.1371/journal.pcbi.1012161)
Supplement: S11 Fig — DN model prediction (red) of the neural response (black) of an example electrode, given a stimulus timecourse (light blue), with (top panel) and without (bottom panel) category-specific scaling. Model prediction for the least preferred image category (faces) results in an overestimation of the neural response, resulting in a strongly negative coefficient of determination (blue arrow). This figure can be reproduced by mkSuppFigure11.py. (PDF) [file pcbi.1012161.s011.pdf]

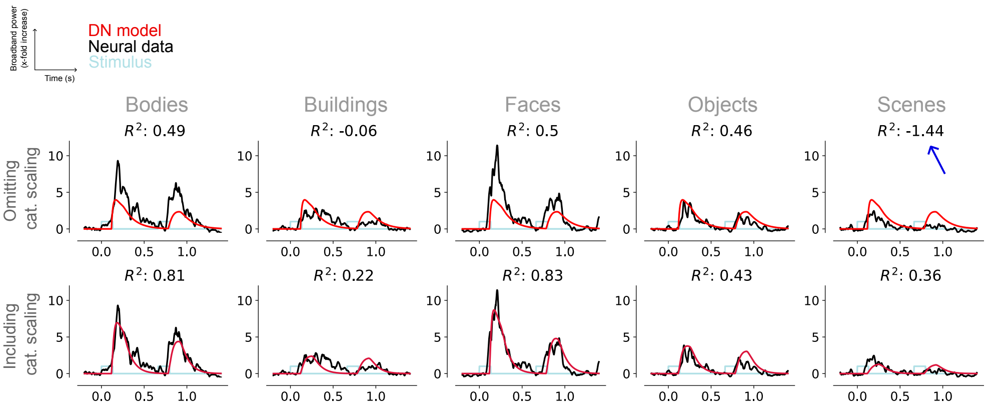

S Fig 11. DN model without category-dependent input strength has poor fits for non-preferred image categories. DN model prediction (red) of the neural response (black) of an example electrode, given a stimulus timecourse (light blue), with (top panel) and without (bottom panel) category-specific scaling. Model prediction for the least preferred image category (faces) results in an overestimation of the neural response, resulting in a strongly negative coefficient of determination (blue arrow).This figure can be reproduced by mkSuppFigure11.py.
