# Supplementary material for: Temporal dynamics of short-term neural adaptation across human visual cortex
Source: PLoS Comput Biol. 2024 May 30;20(5):e1012161. doi: 10.1371/journal.pcbi.1012161 (PMC11166327; doi:10.1371/journal.pcbi.1012161)
Supplement: S1 Table — Columns refer to the following: Subject, subject code in dataset. Age, age of patient at time of recording in years. Sex, gender of the participant. Implantation, type of electrodes implanted. Grid, standard clinical grid; HDgrid, high-density grid; strip, standard clinical strip; depth, depth electrodes. Runs, number of runs where a run is defined as a period of sequential stimulus presentations with no breaks in between. Trials, number of trials collected where half consisted of duration and half consisted of repetition trials (e.g. for sub-p11, there were 432 duration and 432 repetition trials). Repetitions, number of times a stimulus set was repeated (separate stimuli were used for even and uneven runs). (PDF) [file pcbi.1012161.s012.pdf]

| Subject | Age | Sex | Implantation | Runs | Trials | Repetitions |
|---|---|---|---|---|---|---|
| sub-p11 | 19 | F | grid, HDgrid, strip, depth | 6 | 864 | 3 |
| sub-p12 | 42 | M | grid, strip, depth | 4 | 576 | 2 |
| sub-p13 | 22 | F | grid, strip, depth | 4 | 576 | 2 |
| sub-p14 | 32 | F | strip, depth | 2 | 288 | 1 |

**S Table 1. Overview of patient data included in this dataset.** Columns refer to the following: Subject, subject code in dataset. Age, age of patient at time of recording in years. Sex, gender of the participant. Implantation, type of electrodes implanted. Grid, standard clinical grid; HDgrid, high-density grid; strip, standard clinical strip; depth, depth electrodes. Runs, number of runs where a run is defined as a period of sequential stimulus presentations with no breaks in between. Trials, number of trials collected where half consisted of duration and half consisted of repetition trials (e.g. for sub-p11, there were 432 duration and 432 repetition trials). Repetitions, number of times a stimulus set was repeated (separate stimuli were used for even and uneven runs).
