# Supplementary material for: Temporal dynamics of short-term neural adaptation across human visual cortex
Source: PLoS Comput Biol. 2024 May 30;20(5):e1012161. doi: 10.1371/journal.pcbi.1012161 (PMC11166327; doi:10.1371/journal.pcbi.1012161)
Supplement: S2 Table — Columns refer to the following: Visual areas, visual areas to which electrodes are assigned, V1-V3, early visual cortex; VOTC, ventral-occipital cortex; LOTC: lateral-occipital cortex. Matching probabilistic areas, visual areas according to the maximum probability atlas by [42]. Matching retinotopic areas, visual areas according to an anatomically defined atlas by [40] and [41]. (PDF) [file pcbi.1012161.s013.pdf]

Visual area	Matching probabilistic atlas areas	Matching anatomical atlas areas
V1-V3	V1d, V1v, V2d, V2v, V3d, V3v	V1, V2, V3
VOTC	hV4, VO1, VO2	hV4, VO1, VO2
LOTC	TO1, TO2, LO1, LO2, V3a, V3b, IPS	TO1, TO2, LO, V3a, V3b

S Table 2. Overview of visual areas included in this dataset. Columns refer to the following: Visual areas, visual areas to which electrodes are assigned, V1-V3, early visual cortex; VOTC, ventral-occipital cortex; LOTC: lateral-occipital cortex. Matching probabilistic areas, visual areas according to the maximum probability atlas by [42]. Matching retinotopic areas, visual areas according to an anatomically defined atlas by [40] and [41].
