# Supplementary material for: Temporal dynamics of short-term neural adaptation across human visual cortex
Source: PLoS Comput Biol. 2024 May 30;20(5):e1012161. doi: 10.1371/journal.pcbi.1012161 (PMC11166327; doi:10.1371/journal.pcbi.1012161)
Supplement: S3 Table — Columns refer to the following: Subject, subject code in dataset. Electrodes, total number of electrodes. Visual areas, visual areas to which electrodes are assigned, V1-V3, early visual cortex; VOTC, ventral-occipital cortex; LOTC: lateral-occipital cortex. The number of electrodes per area is reported within the parentheses. Matching areas, visual areas included according to the maximum probability atlas by [42] (left column) or a retinotopic atlas developed by [40] and [41] using a Bayesian mapping approach (right column). Visually responsive electrodes, the number of electrodes assigned according by one of the atlases (left column) or assigned manually (right column) to V1-V3, VOTC or LOTC. (PDF) [file pcbi.1012161.s014.pdf]

Subject	Electrodes	Visual areas	Matching areas		Visually responsive electrodes (V1-V3, LOTC, VOTC)	
			Probabilistic atlas	Anatomical atlas	According to atlas	Manually assigned
sub-p11	252	VOTC (4), LOTC (34)	hV4, VO1, TO1, LO1, LO2, V3a, V3b	LO1, LO2, V3b	38	0
sub-p12	76	VOTC (3), LOTC (4)	hV4, VO1, LO1, V3b	hV4, LO2	5	2
sub-p13	116	V1-V3 (3), VOTC (4), LOTC (3)	V2, VO2, TO1, TO2, LO2	V2d, TO1	7	3
sub-p14	94	V1-V3 (14), VOTC (4), LOTC (6)	V2, VO2, TO1, TO2, LO2	V1v, V3d, V3v, hV4, LO1, V3a, V3b	23	1

S Table 3. Overview of electrodes included and visual areas covered in this dataset. Columns refer to the following: Subject, subject code in dataset. Electrodes, total number of electrodes. Visual areas, visual areas to which electrodes are assigned, V1-V3, early visual cortex; VOTC, ventral-occipital cortex; LOTC: lateral-occipital cortex. The number of electrodes per area is reported within the parentheses. Matching areas, visual areas included according to the maximum probability atlas by [42] (left column) or a retinotopic atlas developed by [40] and [41] using a Bayesian mapping approach (right column). Visually responsive electrodes, the number of electrodes assigned according by one of the atlases (left column) or assigned manually (right column) to V1-V3, VOTC or LOTC.
