# Supplementary material for: Temporal dynamics of short-term neural adaptation across human visual cortex
Source: PLoS Comput Biol. 2024 May 30;20(5):e1012161. doi: 10.1371/journal.pcbi.1012161 (PMC11166327; doi:10.1371/journal.pcbi.1012161)
Supplement: S4 Table — Columns refer to the following: d′ threshold, threshold for an electrode to be considered category-selective (see Eq 1, Materials and methods). Image categories, number of electrodes selected per image category. The number of electrodes per area is reported within the parentheses. Total, total number of category-selective electrodes for the specified d′ threshold. (PDF) [file pcbi.1012161.s015.pdf]

| d' threshold | Image categories | | | | | | | | | | Total |
|---|---|---|---|---|---|---|---|---|---|---|---|
| | Bodies | Visual areas | Buildings | Visual areas | Faces | Visual areas | Objects | Visual areas | Scenes | Visual areas | |
| 0.5 | 3 | LOTC | 1 | VOTC | 5 | LOTC (n=3), VOTC (n=2) | 15 | LOTC (n=12), VOTC (n=2), V1-V3 (n=1) | 2 | LOTC | 26 |
| 0.75 | 1 | LOTC | 0 | | 5 | LOTC (n=3), VOTC (n=2) | 5 | LOTC | 1 | LOTC | 12 |
| 1 | 1 | LOTC | 0 | | 4 | LOTC (n=2), VOTC (n=2) | 1 | LOTC | 0 | | 6 |

**S Table 4. Overview of category-selective electrodes.** Columns refer to the following: $d$' threshold, threshold for an electrode to be considered category-selective (see Eq **??**, Materials and methods). Image categories, number of electrodes selected per image category. The number of electrodes per area is reported within the parentheses. Total, total number of category-selective electrodes for the specified $d$' threshold.
