# Supplementary material for: Temporal dynamics of short-term neural adaptation across human visual cortex
Source: PLoS Comput Biol. 2024 May 30;20(5):e1012161. doi: 10.1371/journal.pcbi.1012161 (PMC11166327; doi:10.1371/journal.pcbi.1012161)
Supplement: Attachment — Submitted filename: reviewerLetter.pdf [file pcbi.1012161.s016.pdf]

Dear Editor,

We thank the reviewers for their constructive comments. See below in ***blue*** our responses to the reviewers' comments and associated changes we have made in the manuscript.

We have included additional figures where we use the following referencing:

- **Manuscript Figure**: figures also present in the main manuscript but revised incorporating the reviewer's comments.
- **Additional Figure**: figures only included in this letter to further illustrate our explanations and/or motivations.

Reviewer #1:

The visual world is highly dynamic. In the dynamic realm of visual perception, the temporal characteristics of cortical neurons play an indispensable role. Researchers have developed computational models to more accurately describe how visual cortex neurons encode dynamic information. There are two popular computational models describing the encoding of temporally dynamic perceptual information by populations of visual cortex neurons. (1) The two-temporal-channel (TTC) model (Horiguchi, Nakadomari, Misaki, & Wandell, 2009; Stigliani, Jeska, & Grill-Spector, 2017, 2019) explains the cortical dynamics by the response properties of transient and sustained neuronal populations. (2) The delayed normalization (DN) model (Groen et al., 2022; Zhou, Benson, Kay, & Winawer, 2019), which is built by authors in the current study, tries to explain the temporal dynamics of neural signals in human visual cortex using a unified computational function. The core idea of the DN model is that the stimulus that drives neuronal population activity is divisively normalized by delayed neuronal population activity (delayed via a low-pass filter). The DN model predicts many temporal phenomena in human visual cortex, such as saturation as stimulus contrast and duration increase, suppression for repeated stimuli at short intervals, and slower onsets at low contrast (Groen et al., 2022; Zhou et al., 2019).
The current study builds upon the authors' extensive and commendable efforts in developing the DN model. In a groundbreaking move, Brands and colleagues have integrated a category-selectivity feature module into the original DN model, markedly enhancing its explanatory power. This addition not only elevates the biological interpretability of the DN model but also establishes this work as a pivotal achievement in the realm of visual dynamic modeling.

We thank the reviewer for their positive evaluation of our modeling approach.

However, my appreciation for this research is tempered by certain limitations that I have observed:

Major:
1. In one of the authors' previous works (Groen et al., 2022), an insightful comparison was provided between the DN model and the TTC model. Building on this, I am interested in understanding how the results might differ if the enhanced DN model, now incorporating

category selectivity, were compared to a TTC model that similarly employs a scaling factor for modulating input strength. Such a comparison could offer a more comprehensive understanding of the relative strengths and applications of these two models under modified conditions, and would strongly strengthen the findings in the current study.

Answer:
We appreciate the reviewer's suggestion to expand our modeling to alternative temporal models. To this end, we implemented two instantiations of the two-temporal-channel (TTC) model: one consisting of a linear sustained channel and a transient channel with quadratic nonlinearity (L+Q, Stigliani et al., 2017) and one consisting of a sustained channel with adaptation and a transient channel with sigmoid nonlinearities (A+S, Stigliani et al., 2019). Both models are extended with categorical scaling factors. We provide detailed comparisons with the DN model in the revised manuscript (Supp. Fig 1, see below). Consistent with earlier work (Groen et al., 2022) we find that the DN model better captures iEEG time courses relative to TTC models for the stimuli and temporal manipulations in the current dataset.

The DN model (Supp. Fig 1A, yellow) predicts neural responses more accurately than the L+Q model (Supp. Fig, 1A, green) evident by the significantly better model fits (i.e. cross-validated R^2 values) for V1-V3 and LOTC when applying category-dependent scaling to the stimulus time course for both model types. This is likely due to the fact that the L+Q lacks an adaptation nonlinearity in the sustained channel and is less flexible in the computation of the transient part of the neural response, compared to the DN model.

The DN model also outperforms the more complex A+S model (Supp. Fig. 1A, blue) for electrodes assigned to V1-V3. (We further discuss why electrodes in V1-V3 benefit from adding a category-dependent scaling factor in point 4 below.) For the higher visual regions, the DN model shows numerically higher explained variance than the A+S model but the comparison is not statistically significant. However, we see a qualitative difference in the model's predictions: for longer stimulus durations, the A+S model systematically predicts offset responses that are not always present in the neural responses (Supp. Fig, 1B; we further discuss the offset responses in point 3 below). Moreover, the A+S model overpredicts response widths for VOTC for the longer stimuli (Supp. Fig, 1C) as well as the degree of recovery from repetition suppression, failing to adequately capture the relative differences between lower and higher visual areas for different ISIs (Supp. Fig, 1D). The reason for this overprediction of RS by the A+S model is because this model, as opposed to the DN model, does not include a delayed normalization component that lingers beyond the temporal extent of the impulse response function. Consequently, when the time in between stimuli is sufficiently long, there will not be any effect of previous stimuli on the current response. This results in incorrect predictions for higher visual areas, which show RS even for longer ISIs.

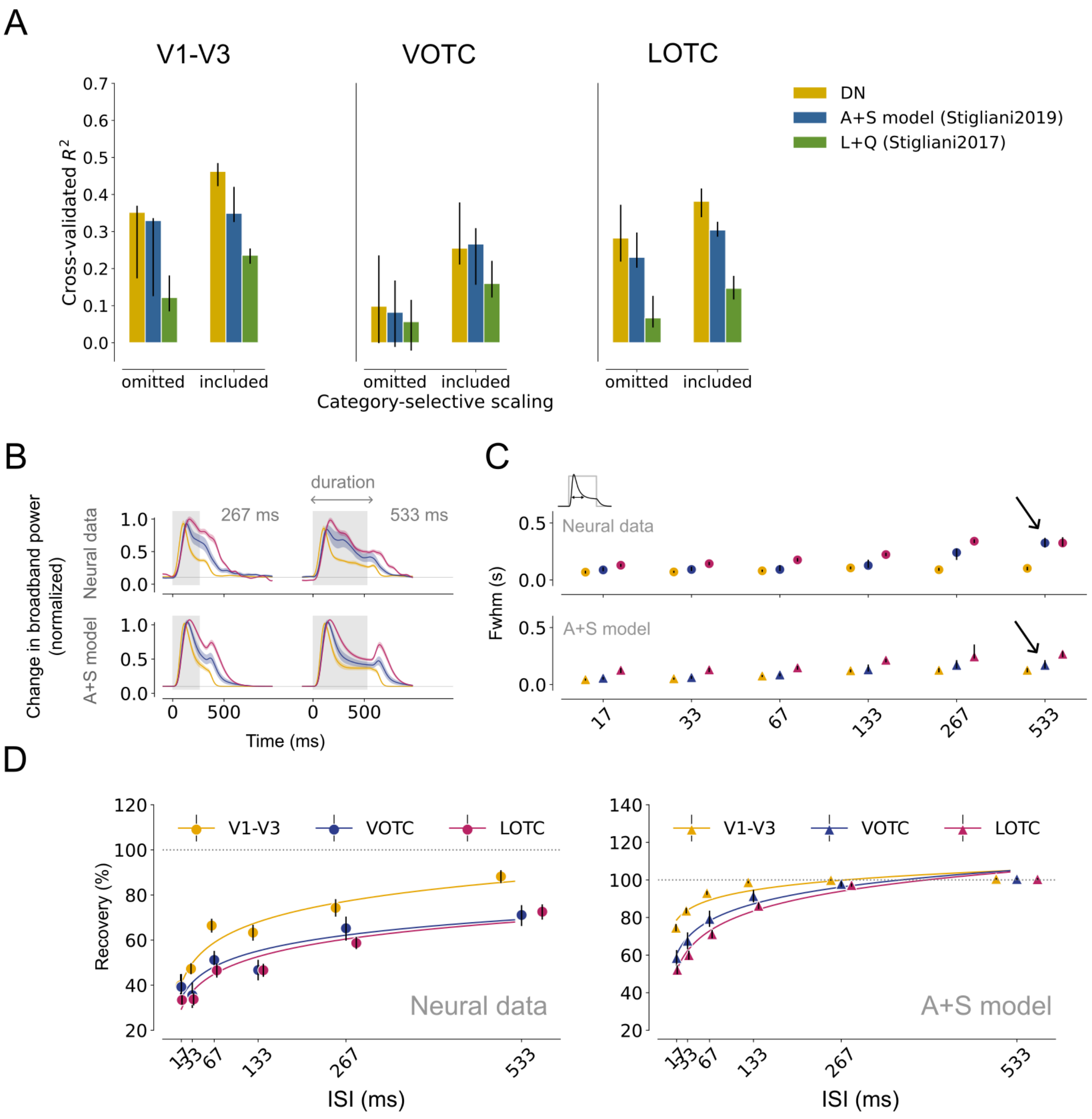

Manuscript Supplementary Figure 1 A: Model fits for the DN model (yellow), the A+S model (blue) and the L+Q model (green). BCD: Prediction of broadband responses by the A+S model (Stigliani et al., 2019) for duration (BC) and repetition (D) trials.

While recent work shows that spatio-temporal versions of TTC models are better predictors of temporal dynamics of fMRI responses (Kim et al., 2023), Groen and colleagues (2022) already noted model failures of the A+S model at predicting iEEG neural time courses, most prominently in the inaccurate prediction of the slower dynamics occurring at lower stimulus contrast. This suggests that the temporal dynamics resulting from interaction of stimulus drive (driven by either contrast changes, or category-selectivity) with neural adaptation are better captured by the normalization dynamics as modeled in the DN model than current TTC models. However, future work should explore why one type of model class appears better for fMRI and the other for iEEG and what the role of experimental design and stimulus manipulations is in this context.

Action:
We now include model comparisons in the Supplementary information (Supp. Fig. 1). Moreover, we report the differences between the models in the Results (page 6 and 9).

2. This study presents an interesting finding that repetition suppression effects differ across preferred and non-preferred image categories. The authors explain these variations in the "Stimulus-specific differences in temporal dynamics in category-selective areas" section of the Discussion, attributing them to a combination of normalization pool component balances and slower normalization dynamics in category-selective areas. However, contrasting observations are noted in the "Higher visual areas exhibit slower and prolonged responses to single stimuli" section and Supplementary Figure 1, showing no such variations in transient-sustained dynamics. The authors propose that this could be due to inherently slower temporal integration in the transient-sustained dynamics of higher visual regions. Given these observations, it would be beneficial for the authors to provide a more detailed explanation of how these findings align within a unified theoretical framework. Particularly, it would be insightful to explore how category selectivity influences the two forms of temporal adaptation differently. Expanding on this point in the discussion, possibly including a comparative analysis with findings from non-human primate studies, could significantly deepen and broaden the scope of the current study's conclusions.

Answer:
Here, the reviewer asks whether we can offer an explanation for our finding that stimulus preference does not affect transient-sustained dynamics for single stimuli but that it does affect recovery from repetition suppression for repeated stimuli.

For repeated stimuli, we indeed find clear evidence of an effect of stimulus preference on repetition suppression. The DN model provides an explanation by suggesting that the stronger RS for preferred stimuli arises due to lingering activity of the normalization pool at the start of the second response interacting with the input drive differences reflecting stimulus effectiveness (which differ most strongly between preferred and non-preferred stimuli in category-selective areas; see Figure 9).

For single stimuli, the DN model does in fact predict slightly different temporal dynamics for preferred vs non-preferred stimuli. This is evident in the model predictions, which show a more transient response to preferred stimuli, paralleling our earlier findings comparing high vs. low contrast (Groen et al, 2022). This pattern can also be seen in the data (Additional Figure 3, shown below as well). However, this effect is small - and dwarfed by the overall differences in dynamics between lower and higher visual regions - and the derived metrics (i.e. time to peak and full-width at half maximum) did not significantly differ between areas. We therefore decided not to report this as a finding in the manuscript.

Additionally, we followed the reviewer's suggestion to revisit prior literature to see if our observed stimulus preference effects were consistent with repetition suppression and transient-sustained dynamics observed in non-human primates. Consistent with our findings,

several such studies indeed report that stimulus preference affects repetition suppression on the single-cell or neural population level, but none report transient-sustained differences in single-stimulus responses. This could either be because these effects were weak or absent, or because the authors did not test for them; all we know is that these prior studies lack detailed analysis of single-stimulus response dynamics and did not use a computational model (delayed normalization or otherwise) to predict temporal dynamics. We now report this comparison in our revised Discussion section.

Action:
We mention the stimulus-specific differences for higher visual areas in the Results section (page 6/7) and, following the suggestion of the reviewer, we provide a more in-depth comparison with data collected in non-human primates in the Discussion (page 16).

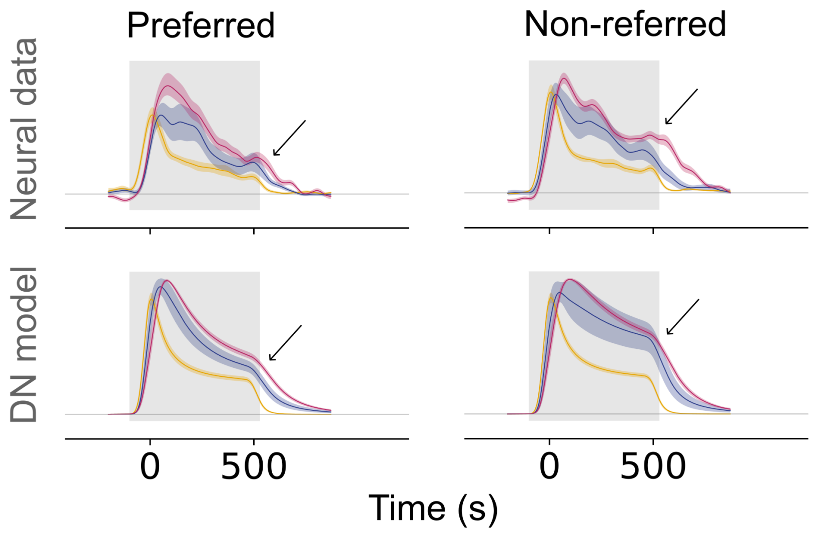

Additional Figure 3 Broadband (top) and model (bottom) time courses for V1-V3 (yellow), VOTC (blue) and LOTC (pink) for preferred (left) and nonpreferred (right) stimuli. The model predicts a slightly more transient response for preferred stimuli, which also appears to be present in the neural data, but the differences are relatively subtle.

3. The neuronal response to stimulus offset is a significant aspect of neurophysiological studies, as evidenced by relevant research such as Stigliani et al. (2019), which highlighted the importance of offset response. In this context, I noticed that the current study does not include an analysis of the offset response, either in the neural or model responses. Could the author provide an explanation for this omission? Understanding the rationale behind excluding the offset response would not only clarify the scope of the study but also help in comprehensively evaluating its findings in the context of existing literature.

Answer:
The reviewer asks us why we did not include any analyses of offset responses. The main reason for this is that we simply do not observe many offset responses in our iEEG data (we already

briefly remarked on this in our original Discussion, page 16). In fact, we find that only < 10 out of a total of 79 electrodes showed an offset response, and as noted above, the lower performance of the TTC model seems to be partly due to it predicting offset responses when these are not present in the data. A similar lack of offset responses was observed in earlier iEEG studies (Groen et al., 2022, Zhou et al., 2019).

To illustrate this, below the broadband time courses for the electrodes assigned to VOTC are plotted (Additional Figure 4). Here it can be observed that most of the electrodes do not show an offset response. We observed a similar trend for the electrode groups assigned to V1-V3 and LOTC.

We do not have a definitive explanation for this lack of offset responses. Zhou et al. (2019) noted that offset responses were more pronounced for electrodes with peripherally tuned spatial receptive fields (beyond 5 degrees eccentricity). The stimuli in our current experiment extended to 8.5 eccentricity, therefore the lack of offset responses may be related to the spatial coverage of the stimulus. However, other explanations are possible, in particular how the neural signal is sampled using fMRI (in which offset responses are commonly observed, e.g. Stigliani et al., 2019) versus iEEG.

Action:
We expanded our Discussion section (page 16) where we discuss the lack of offset responses in more depth.

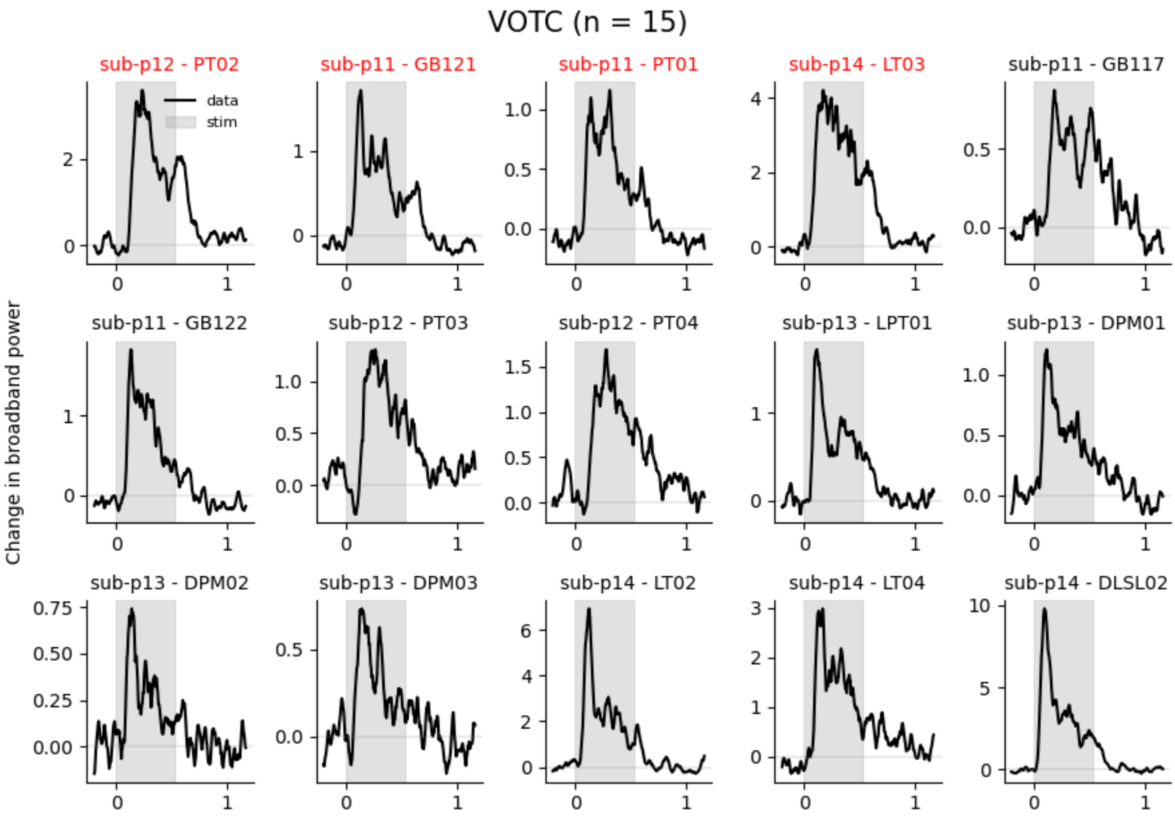

Additional Figure 4 Broadband time courses for a duration trial plotted per electrode in VOTC. For the electrodes depicted in red an offset response is visible.

4. It's well-documented that the V1-V3 areas respond to stimuli differently compared to higher visual areas. Specifically, while higher visual areas show significant category selectivity, the V1-V3 areas are more attuned to low-level feature selectivity. Given this distinction, I am keen for the authors to clarify how integrating a category-selectivity-based scaling factor into the model's input strength specifically benefits its performance in the V1-V3 regions.

Answer:
Here, the reviewer asks us to clarify why adding a category-based scaling factor results in better fits in early visual cortex, which is not typically considered to exhibit category-selectivity. We attribute this to covariation of low-level feature differences with the categories in the dataset. Specifically, one of the six categories consisted of scrambled stimuli which had many edge elements, and another of scene stimuli that had a slightly larger retinotopic extent than the other classes which consisted of cropped objects. These classes likely drive responses in early visual areas better compared to the other stimulus classes, resulting in a benefit of class-specific stimulus scaling.

Indeed, if we inspect the responses in V1-V3 for the different classes and associated scaling factors estimated by the model, we see that scrambled and scene classes yield on average more activation (Additional Figure 5). We expect that such differences may not be present if a more controlled stimulus set would be used and thus a category-scaling would not have much benefit for modeling V1-V3.

Action:
We added a sentence to the Discussion (page 16) in which we clarify why we think our category-scaling benefits explaining neural response dynamics in V1-V3 even though these regions are not considered category-selective.

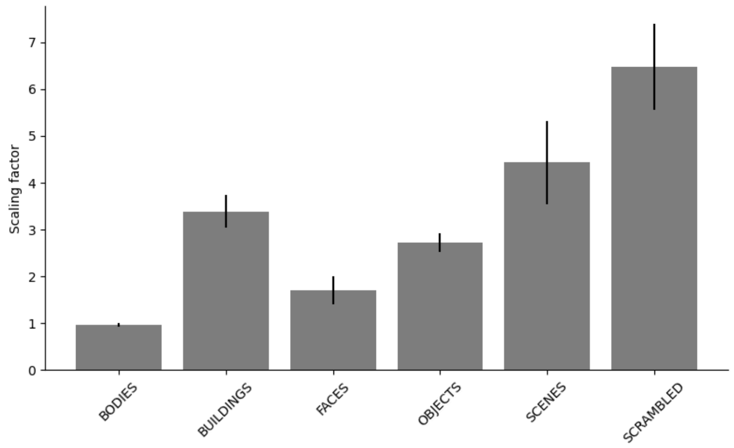

Additional Figure 5 Category-dependent scaling factors averaged over all electrodes in V1-V3. Neural responses show strongest selectivity for images depicting buildings, scenes and scrambled patches evident by the higher scaling factor values.

Moreover, it would be insightful if the study examined the potential enhancement of the model's predictive performance through the inclusion of low-level feature selectivity parameters, such as orientation or color. An exploration in this direction could provide a more comprehensive understanding of the model's effectiveness across various visual processing areas and its capability to encompass a wider range of neuronal responses.

Answer:
Here, we incorporated category-selectivity in the model by adding a single scaling parameter for each image category. While we agree with the reviewer that extending our modeling framework to incorporate tuning to low-level spatial properties such as orientation and color is worthwhile, this poses a challenge in our current dataset of naturalistic stimuli because orientation and color space can be parameterized in many different ways. One idea could be to extend the one-dimensional input and add several parameters each describing a different low-level stimulus aspect. To properly develop and evaluate such a model however would require a larger stimulus set in which the different low-level features are more controlled.

5. The study currently does not report the specific number of category-selective electrodes present in the VOTC and the LOTC. For a more comprehensive understanding of the analysis, it is crucial to have detailed information regarding the proportion of category-selective electrodes in VOTC and LOTC.

Action:
We apologize for this omission and have updated Table 4 (page 29) by reporting per visual region the number of category-selective electrodes.

6. In the Materials and Methods section, the authors outline "two consecutive data selection steps". These steps include an initial normalization of the signal to a percentage signal change, which is then followed by determining responsiveness using a z-score threshold, effectively serving as a second normalization process. Could the authors elaborate on the reasoning behind the use of two different normalization techniques in this sequential manner?

Answer:
Our apologies for the unclear formulation of this part of the analysis. Converting responses to z-score is only applied during the electrode selection procedure. The figures in the Results section show the responses after applying only the initial normalization (i.e. computing the x-fold increase from baseline). The reason for applying the z-score during electrode selection is because response magnitudes when expressed as an x-fold increase in power vary to a high degree across electrodes. To determine the response onset latency, we were interested in the relative increase after presenting the stimulus, and for this reason time courses were converted to a z-score.

Action:

We have clarified the logic and procedure in the revised manuscript (page 20).

7. The manuscript lacks detailed explanations on how the scaling factor is computed.

Action:
We now more clearly outline the implementation and rationale of the scaling factor computation (page 22).

Furthermore, the rationale behind applying the scaling factor to the model's input strength, as opposed to its output, is not clearly articulated. This aspect raises questions, especially since category selectivity is typically associated with later stages in the visual hierarchy.

Answer:
The reviewer justly comments that it is not trivial to determine at what stage of visual processing category-dependent scaling occurs. However, scaling the output (i.e. predicted neural time course) results in a failure to predict the stimulus-specific effects for category-selective electrodes (Additional Figure 6). When the output is scaled, a linear transformation is applied, resulting in a proportional scaling of the response. In this case, the general response shape is not affected and therefore the model predicts the same amount of RS for both preferred and non-preferred stimuli. On the other hand, scaling the stimulus time course, followed by the model computations, allows for factoring in the effect of stimulus category on neural responses in a nonlinear manner, where the varying heights of the different stimulus categories result in a distinct balance between the normalization pool components, and therefore predicts different degrees of RS for preferred and non-preferred stimuli, which aligns with what we observe in the neural data.

Scaling the input stimulus is of course a highly simplified way of modeling differences in stimulus drive, especially for electrodes located in areas associated with later stages in the visual hierarchy, which receive their inputs from previous areas that already apply nonlinear transformations to the input. A possible extension of our modeling approach in this direction would be to use models that cascade input-output transformations mimicking the neural processing from lower- to higher-level areas (as briefly explored in Zhou et al., 2019, their Fig. 7C), or in deep neural network models that incorporate divisive normalization. We hope that making our data openly available on OpenNeuro will facilitate the development and benchmarking of such models against neural data.

Action:
We expanded our Discussion section (page 17) where we discuss future directions including models that mimic the information processing from lower to higher visual areas in a more realistic way.

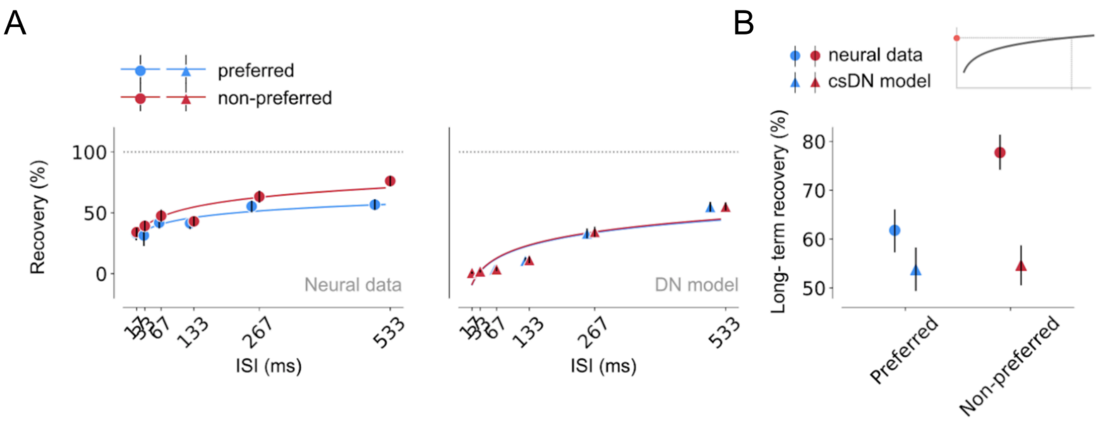

Additional Figure 6 A: Recovery from RS as a function of the ISI derived from the neural data (left) and model predictions (right) for preferred (blue) and non-preferred (red) stimuli. B: Long-term recovery computed as the degree of recovery from RS for an ISI of 1 s. When scaling is applied on the predicted neural responses, the DN model predicts identical response shapes and long-term recovery for preferred and non-preferred stimuli which does not align with the neural data.

Minor:
1. In Figure 7 & 9, top panel, "normalisation" should be "normalization".

Action: Revised Figure 7 and 9 in manuscript.

2. On page 14, line 7, "Differences in temporal dynamics between for ventral and lateral occipital cortex" should be "Differences in temporal dynamics between ventral and lateral occipital cortex".

Action: Corrected text.

3. On page 4, in the third paragraph, "retonotopic atlases" should be "retinotopic atlases".

Action: Corrected text.

4. In Figure 6B and Supplementary Figure3, "AVerage ecovery (%)" should be "Average recovery (%)"

Action: Revised Figure 6 in manuscript.

5. The full form of the abbreviation "csDN" is not provided in the manuscript.

Action: Introduced abbreviation on page 22.

References:
Groen, I. I., Piantoni, G., Montenegro, S., Flinker, A., Devore, S., Devinsky, O., . . . Ramsey, N. F.

(2022). Temporal dynamics of neural responses in human visual cortex. Journal of Neuroscience, 42(40), 7562-7580.
Horiguchi, H., Nakadomari, S., Misaki, M., & Wandell, B. A. (2009). Two temporal channels in human V1 identified using fMRI. Neuroimage, 47(1), 273-280. doi:https://doi.org/10.1016/j.neuroimage.2009.03.078
Stigliani, A., Jeska, B., & Grill-Spector, K. (2017). Encoding model of temporal processing in human visual cortex. Proceedings of the National Academy of Sciences, 114(51), E11047-E11056.
Stigliani, A., Jeska, B., & Grill-Spector, K. (2019). Differential sustained and transient temporal processing across visual streams. PLoS Computational Biology, 15(5), e1007011. doi:10.1371/journal.pcbi.1007011
Zhou, J., Benson, N. C., Kay, K., & Winawer, J. (2019). Predicting neuronal dynamics with a delayed gain control model. PLoS Computational Biology, 15(11), e1007484.

Reviewer #2:

This interesting study shows that neural adaptation differs across human visual cortex and suggests that the underlying neural computations can be explained by a model of delayed divisive normalization.

However, I have major concerns that need to be addressed.

We thank the reviewer for their feedback. The expressed concerns touch upon several important aspects regarding the neural circuits underlying the observed neural dynamics (e.g. excitation/inhibition ratios, parvocellular vs magnocellular input). However, we believe our dataset and computational model might not be suited to investigate many of these aspects.

We would like to emphasize that our model explanations are agnostic regarding neural processes that occur on the single-cell level. Instead, they provide insights into how population activity (arising from a stimulus presentation) and history-dependent normalization (implemented as temporal divisive normalization) could elucidate observed differences in adaptation across multiple brain regions and stimuli. The DN model is not a circuit-level model, and the predicted responses may arise from a multitude of biophysical and cellular mechanisms. Identifying neural circuitry that could give rise to normalization dynamics will require alternative types of data, such as single-cell recordings that we do not have access to in our human iEEG patients. To clarify this, we have added new text addressing this aspect of our study and the modeling approach in the Discussion section (page 17). Below we further address the reviewer's individual comments.

Other forms of normalization are viable. In particular a comparison with other alternative models of normalization is critical. Otherwise it is difficult to ascertain whether a single type of model is viable.

Answer:
The reviewer's suggestion to expand our modeling to alternative temporal models is appreciated. In response to Reviewer 1's comments above, we have performed additional

analyses and compared DN model fits with a previously developed model which predicts neural responses using distinct channels, responsible for either the transient and sustained responses observed in neural signals, known as the two-temporal channel (TTC) model (Stigliani et al., 2017, 2019). We show that the DN model outperforms the TTC model in Stigliani et al. (2017) and offers a qualitatively better fit compared to the TTC model in Stigliani et al. (2019). For more detail, see point 1 of reviewer 1.

The authors also discuss fatigue and sharpening mechanisms but do not make explicit comparisons.

Action:
We agree with the reviewer's comment regarding the lack of comparison of the DN model with fatigue and sharpening mechanisms and have therefore decided to remove this section of the discussion in the revised manuscript.

It is important to discuss the results in terms of excitation/inhibition ratios and principles of organization of the visual cortex.

Answer:
As we mentioned above, the DN is not a circuit-level model and therefore agnostic about the interaction and connectivity of the underlying excitatory and inhibitory neural populations. We therefore do not discuss single/multiple-cell dynamics, including excitation/inhibition ratios, in detail. However, in the discussion we refer to earlier publications on the DN model (e.g. page 14, Zhou et al., 2019) and also mention a review paper in the Materials & Methods section in which putative roles of glutamatergic and GABAergic connections in divisive normalization mechanisms are discussed (page 22, Carandini & Heeger, 2012).

Levels of parvocellular and magnocellular input are critical for interpretation of adaptation mechanisms and they are not addressed.

Answer:
The new comparisons with two-temporal channel (TTC) models addresses this point a little bit, since the two-channel implementation is inspired by classic observations showing that the magnocellular (M) pathway exhibits more transient responses and the parvocellular (P) pathway more sustained responses (Stigliani et al., 2019). We however observed a qualitative poorer fit by the TTC model compared to the DN model, suggesting that modeling distinct distributions of parvocellular and magnocellular inputs does not result in better explanation of the adaptation observed in our current dataset of iEEG neural responses. We believe this is partly due to the fact that few electrodes in our dataset show offset responses, which are explicitly modeled in the TTC model by the transient channel. (For further details see point 1 and 3 of reviewer 1). Moreover, previous studies point towards evidence of mixed magno- and parvocellular pathways instead of isolated streams of processing throughout the cortical hierarchy (for a review see Callaway, 2005). It is therefore unclear how these different pathways contribute to the transient and sustained parts of the neural response.

The same holds true for dorsal-ventral asymmetries in adaptation.

Answer:
Dorsal-ventral asymmetries in adaptation are addressed to some extent by our grouping of higher visual cortex electrodes into VOTC (i.e. ventral cortex) and LOTC (lateral cortex). For single stimulus responses, our results suggest similar response rises (Fig. 4B) and broader response widths for some stimulus durations for LOTC (Fig. 4C). For repeated stimuli, we observe similar degrees of and recovery from RS for VOTC and LOTC (Fig. 6). We reflect on these findings in the Discussion (page 15/16) where we discuss in more detail previous work and putative roles differences in temporal dynamics ventral and lateral streams in visual cortex.

The authors could consider hysteresis signatures as a way to address adaptation mechanisms.

Answer:
Hysteresis has mostly been studied in the context of moving stimuli (Schwiedrzik et al, 2012; Sayal et al., 2020) or varying contrast conditions (Kleinschmidt et al., 2002) while behavior was measured. While we agree hysteresis is an interesting concept and worthwhile investigating in the context of adaptation, we believe that our stimulus set (static images with similar contrasts) and experimental design (i.e. trial types were presented randomly) are not appropriate to investigate this. Moreover, the participants were performing an orthogonal task which makes it challenging to relate the neural responses to behavior.

The level of precision of identification of visual areas is quite low and could be improved.

Answer:
We are uncertain how to interpret this concern. To identify visual areas, we use probabilistic retinotopic atlases, similar to our previous work (Groen et al., 2022; Yuasa et al., 2023). Obtaining functional mapping (using fMRI) that would allow to identify visual areas in these iEEG patients is typically not feasible due to their clinical condition; this and our previous studies are to our knowledge the among first ones to have developed analysis pipelines to relate electrode position to retinotopic atlases allowing a (probabilistic) assignment of electrodes to (putative) visual maps.

If the reviewer is criticizing our division of electrodes to three relatively coarse groups (V1-V3, VOTC and LOTC), we choose for this coarse division due to the clinical nature of the electrode positions in iEEG recording and the lack of control over where the locations were placed and because we are aware that our visual area assignment is in fact probabilistic. Making a more fine-grained division of electrodes would result in very few electrodes per area and potentially exacerbate subject biases.

Stimulus durations are very short (maximum 533 ms) thereby excluding the study of adaptation of other relevant temporal scales.

Answer:

Our study explicitly focuses on short-term adaptation (see Introduction, paragraph 2), extending on earlier work studying the neural processes underlying short-term adaptation (Zhou et al., 2019; Groen et al., 2022). While we agree long-term adaptation is an important aspect of temporal processing, we believe this is a separate research direction, especially when considering previous findings which has shown short- and long-term adaptation employ different neural circuits and strategies (Miller & Desimone, 1994; Pavan et al., 2010; Patterson et al., 2013).

Action:
Throughout the manuscript, we have indicated more clearly that our study focuses on short-term adaptation only.

The authors also ignore at this point important single cell studies on adaptation

Answer:
We thank the reviewer for the suggestion and would welcome concrete pointers on which studies to include. We currently cite several single-cell studies, including Miller & Desimone (1991, 1994), Sobotka & Ringo (1994) and Sawamura et al. (2006).

Biological plausibility of parameter differences across the visual hierarchy is not adequately justified. The model differences in temporal normalization dynamics have little biological justification. This needs to be improved

Answer:
The DN model is a delayed gain model describing what canonical computations need to be applied to a one-dimensional stimulus time course in order to transform this time course into a neural response time course prediction that captures the temporal dynamics (shape) of actual observed neural population responses in human visual cortex. The different parameters model a history-dependent normalization process which determine several aspects of the response shape such as the width of the transient after stimulus onset and the decay of the response. In other words, the parameters characterize the input-output behavior of the model. While individual components of canonical divisive normalization models are thought to correspond to specific neural processes such as voltage to spiking transform (in the case of the exponentiation; see Zhou et al., 2019; Caradini & Heeger et al., 2012), here the parameter values are obtained using an empirical approach and are not directly linked to neural processes occurring on the single-cell level.

Increased temporal windows across the cortical hierarchy may indeed have several computational benefits but this is not necessarily true in some pathways.

Action:
We did not intend to attribute increasing temporal windows across cortical hierarchy solely to computational benefits. We have formulated our explanations for the observed increasing time windows across the visual hierarchy more carefully in the Discussion (page 15).

—---

References:

Callaway, E. M. (2005). Structure and function of parallel pathways in the primate early visual system. *The Journal of physiology*, *566*(1), 13-19.

Miller, E. K., Li, L., & Desimone, R. (1993). Activity of neurons in anterior inferior temporal cortex during a short-term memory task. *Journal of Neuroscience*, *13*(4), 1460-1478.

Patterson, C. A., Wissig, S. C., & Kohn, A. (2013). Distinct effects of brief and prolonged adaptation on orientation tuning in primary visual cortex. *Journal of Neuroscience*, *33*(2), 532-543.

Pavan, A., Campana, G., Maniglia, M., & Casco, C. (2010). The role of high-level visual areas in short-and longer-lasting forms of neural plasticity. *Neuropsychologia*, *48*(10), 3069-3079.

Schwiedrzik, C. M., Ruff, C. C., Lazar, A., Leitner, F. C., Singer, W., & Melloni, L. (2014). Untangling perceptual memory: Hysteresis and adaptation map into separate cortical networks. *Cerebral Cortex*, *24*(5), 1152-1164.

Sayal, A., Sousa, T., Duarte, J. V., Costa, G. N., Martins, R., & Castelo-Branco, M. (2020). Identification of competing neural mechanisms underlying positive and negative perceptual hysteresis in the human visual system. *NeuroImage*, *221*, 117153.

Kleinschmidt, A., Büchel, C., Hutton, C., Friston, K. J., & Frackowiak, R. S. (2002). The neural structures expressing perceptual hysteresis in visual letter recognition. *Neuron*, *34*(4), 659-666.

Groen, I. I., Piantoni, G., Montenegro, S., Flinker, A., Devore, S., Devinsky, O., ... & Winawer, J. (2022). Temporal dynamics of neural responses in human visual cortex. *Journal of Neuroscience*, *42*(40), 7562-7580.

Yuasa, K., Groen, I. I., Piantoni, G., Montenegro, S., Flinker, A., Devore, S., ... & Winawer, J. (2023). Spatial Tuning of Alpha Oscillations in Human Visual Cortex. *bioRxiv*, 2023-02.

Reviewer #3:

Brands and colleagues showed natural stimuli of varying duration, as well as two stimuli with varying inter-stimulus interval (ISI), to study the effect of adaptation and repetition suppression (RS) in patients with iEEG electrodes in early and late visual areas. They found that higher areas have slower and more pronounced adaptation to single stimuli, as well as slower recovery in the RS paradigm. They used a delayed divisive normalization (DN) model to explain their finding.

I think the question is well motivated and their experimental paradigm and analyses to be thorough overall (except a few issues as detailed below).

We thank the reviewer for their positive evaluation of our question, experimental paradigm and analyses.

However, I was also not surprised by these results, and therefore unsure about the novelty and significance of these results in the broader context. My main issues are as follows:

1. Since higher visual areas have higher latency to visual stimulation, and these natural stimuli are expected to produce variable responses in these areas that are involved in feature integration etc, it is not surprising that the DN model with a large delay parameter explains these results (Figure 4).

Answer:
We appreciate the reviewer's comment. We would like to clarify that in our fitting procedure, we include onset latency as a nuisance parameter, meaning that simple delays of the response onset due to the visual signal 'arriving later' in higher visual areas is not included in the model fits. Instead, the DN model captures differences in temporal dynamics in the response itself. Moreover, the delay parameter in the DN is not a delay in the response, but in the normalization, determining the shape of the response. Our results clearly show that both the rise and decay of the neural population response is slower in higher visual areas (not just the response latency). While it is perhaps indeed to be expected that, presumably due to additional computational complexity, responses in these regions are less precisely 'aligned' to the stimulus onset, it is not trivial that a delayed normalization model should accurately capture the precise dynamics of the response.

2. I feel the more nuanced results related to how normalization acts were not clearly visible in the raw data and could be due to the scaling of responses to 1. Most striking case is Figure 8A, where the preferred and non-preferred (blue and red traces) conditions are completely overlapping for neural data (top row) but show systematic differences in the DN model (bottom row). Indeed, this scaling could be an issue. If the actual response is weak beyond the noise floor, the model may try to fit it in a different way, as suggested by the authors in Figure 9. But this noise floor in the iEEG signal could be due to factors that are independent of the normalization pool. I think it would be good to show non normalized responses in some cases to make this point more explicit.

Answer:
We appreciated the reviewer's concern relating to the visualizations of the category-specific effects. Please note that this does not affect response fits along the lines suggested by the reviewer, as all model fits were done on non-normalized data (see model fitting procedure described on page 21); in other words, peak-normalization was applied only for visualizing response and model prediction time courses and their associated dynamics in Figures 4, 5 and 8. The motivation to show peak-normalized responses is that they allow for better inspection of the dynamics (shape) of the responses independent of the magnitudes. These differences are more difficult to see when plotting non-normalized responses (see Additional Figure 7 below). For full transparency, we have now added this visualization to Supplementary materials.

Moreover, to ensure that potential differences in signal would not affect model fits and that the non-preferred trials had sufficient SNR, we considered the non-preferred stimuli as the average over all the image categories for which an electrode showed least selectivity (i.e. every image category except for the preferred category). The SNR (z-score = mean/std) showed 0.52 and 0.54 for time courses for preferred and non-preferred image categories, respectively, which is considerably higher than the set threshold that was used to select electrodes for the analysis focusing and low vs. high visual areas (V1-V3, VOTC and LOTC), which was 0.2.

Action:
We have added Additional Figure 6 to the Supplementary Information (page 35).

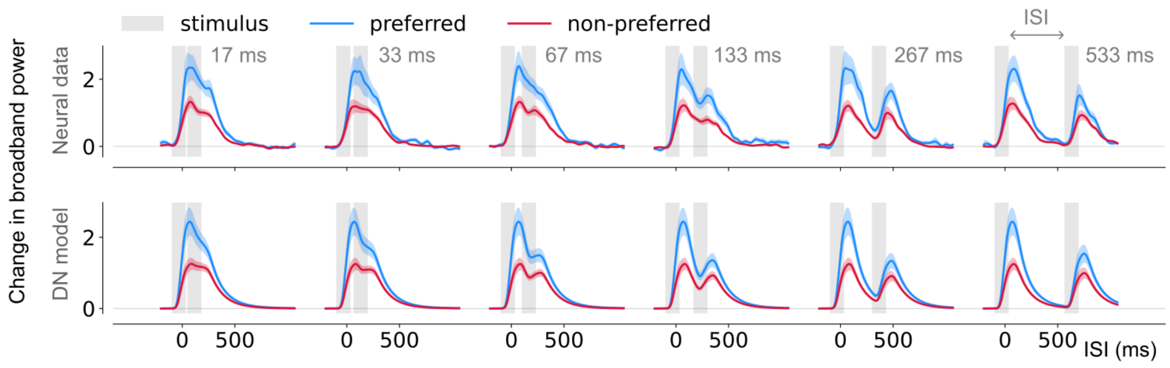

Additional Figure 7 Top**,** Broadband time courses for preferred (blue) and non-preferred (red) stimulus trials plotted separately for each ISI. Bottom, Same as the top panel for DN model predictions.

3. Likewise, the promised stronger recovery from adaptation for lower areas as shown in Figure 5B (yellow versus red) is not visible in the raw traces in Figure 5A, where both red and yellow reach almost the same level for the second stimulus for the ISI of 533 ms.

Answer:
We agree that the stronger recovery from adaptation in early visual areas is not easily identifiable in the raw traces in Fig 5A. As we highlighted in our manuscript it is important to subtract the response of the first stimulus to visualize these effects (this is shown in Fig 5B). In addition, our metric of recovery as reported in Fig. 6A is the area under the curve (AUC) for the entire response (Fig 8A). The reported stronger recovery for lower areas is only partly due to the peak magnitude of the response. Another aspect that strongly contributes to the area-wise differences is the reduced response after the peak for higher visual areas (see Additional Figure 8 below, pink shaded regions for VOTC and LOTC).

Action:

We have noted in the Result section (page 8/9) that part of the area-specific differences in degree of RS are present in the response decay rather than the peak response magnitude and updated Fig. 5B in the main manuscript to point out the area-specific differences more clearly.

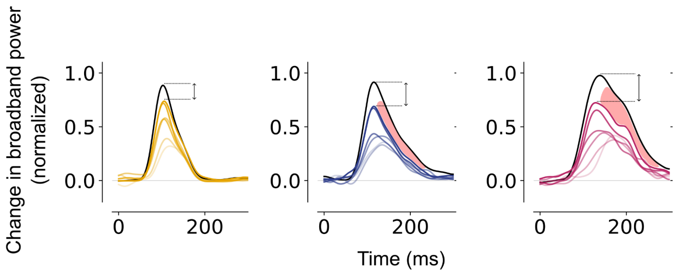

Additional Figure 8 Estimated, normalized response to the second stimulus for V1-V3 (yellow), VOTC (blue) and LOTC (pink). The response to the first stimulus is shown in black.

4. The number of subjects in each visual category should be mentioned upfront in the Results. It appears that majority of data comes from two subjects only.

Action:
We have updated Table 3 (page 29) and reported the number of electrodes found per subject and visual area. The fact that some patients disproportionally contribute to the dataset is largely due to the presence of a high-density grid in patient sub-p11. While this patient indeeds contributes most data to LOTC, all three visual areas include electrodes from multiple patients.

Minor: Figure 6B y-axis should read Average recovery. ‘r’ is missing.

Action: Revised Figure 6 in manuscript.
