# Supplementary material for: Temporal dynamics of short-term neural adaptation across human visual cortex
Source: PLoS Comput Biol. 2024 May 30;20(5):e1012161. doi: 10.1371/journal.pcbi.1012161 (PMC11166327; doi:10.1371/journal.pcbi.1012161)
Supplement: Attachment — Submitted filename: letterReviewer.pdf [file pcbi.1012161.s018.pdf]

Dear Editor,

We thank the reviewer for the constructive comments. See below in ***blue*** our responses to the reviewer's comments and associated changes we have made in the manuscript.

Apart from the changes made in response to the reviewer's comment, we also fixed a small mistake in the naming of the atlases in the Supplementary Tables 2-3, and we have updated our Data and Code availability statement in order to comply with PLOS CB guidelines.

Comments for Brands et al., Temporal dynamics of short-term neural adaptation across human visual cortex (PCOMPBIOL-D-23-01549R1).

The revised manuscript has been carefully examined. I recognize the significant contributions this work presents. However, there remain a few nuanced areas that would benefit from additional refinement.

1. In the abstract, the author concludes that the study revealed temporal adaptation across the human visual hierarchy. However, upon review of Supplementary Table 3, it is evident that there is an imbalance distribution of electrodes among brain areas. This imbalance raises concerns regarding the sufficiency of using the term "visual hierarchy" as it is plausible that the unevenly distributed electrodes across brain areas may not adequately represent the entirety of the visual hierarchy. It is suggested that a more cautious interpretation could be considered to improve the validity of the findings.

Answer: We appreciate the reviewer's suggestion to interpret the results regarding differences in dynamics across the visual hierarchy with more caution. Supplementary Table 3 shows the distribution of electrodes is imbalanced in two ways: 1) the three visual areas we considered did not contain the same number of electrodes, meaning that summary statistics and parameter estimates could be more robust for one area than another; and 2) subjects contributed different numbers of electrodes to each area, meaning area differences could partly reflect subject differences.

Regarding the first point, we already mention in the Discussion that because electrode placement was clinically determined, we chose to use coarse groupings of areas (one lower and two higher groups, corresponding to the ventral and lateral surfaces of visual cortex) and acknowledged that for finer-grained differences across the hierarchy, more data is required. We furthermore performed all analyses (e.g. computation of responses shape parameters such as time-to-peak, or computing DN model fits) at the single-electrode level, allowing for inspection of these properties for individual electrodes in a given area and estimation of variability within areas, shown in confidence intervals and error bars shown in our figures.

Regarding the second point, to verify the robustness of the area differences as presented in the paper, we have plotted the responses to single stimuli separately for the two individual subjects who had electrode coverage for all three visual maps (V1-V3, VOTC and LOTC, **Additional Figure 1**). Responses for electrodes assigned to V1-V3 have narrower response widths compared to VOTC for sub-p13 (**Additional Figure 1A**) and VOTC and LOTC for sub-p14 (**Additional Figure 1B**), suggesting that the observed faster temporal dynamics in early visual areas compared to later visual areas robust across both subjects. However, the

relatively fast responses in LOTC for sub-p14 suggests there is some individual variability in these aspects.

Action: We have expanded our Discussion section on limitations of our study, adding an explicit reference to the uneven distribution of electrodes, and the possible influence of subject effects. Moreover, we have adapted the abstract, author summary, introduction and discussion by omitting the term ‘visual hierarchy’ whenever we are describing our findings, using the term ‘differences between areas’ exclusively.

A

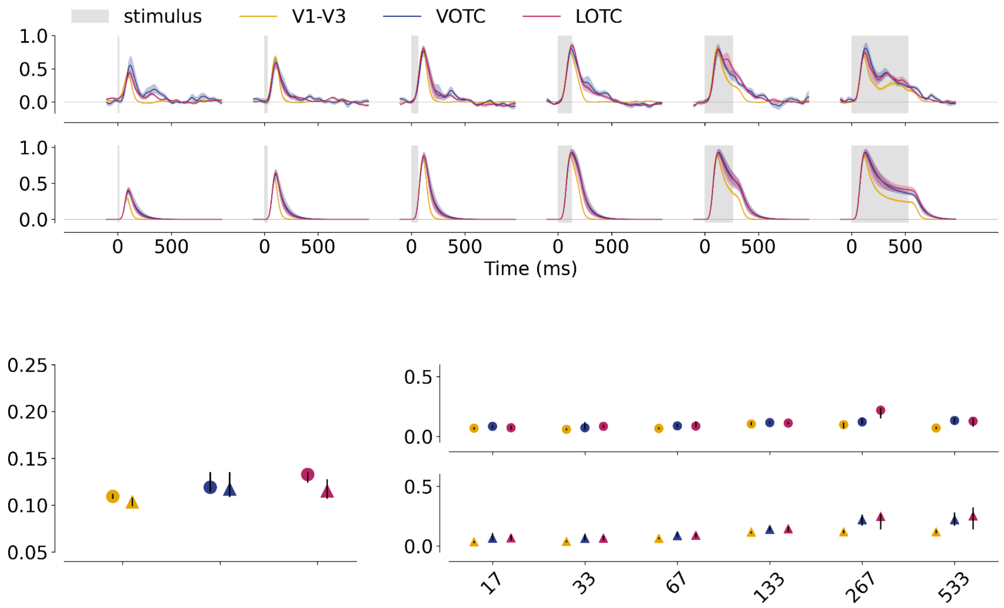

B

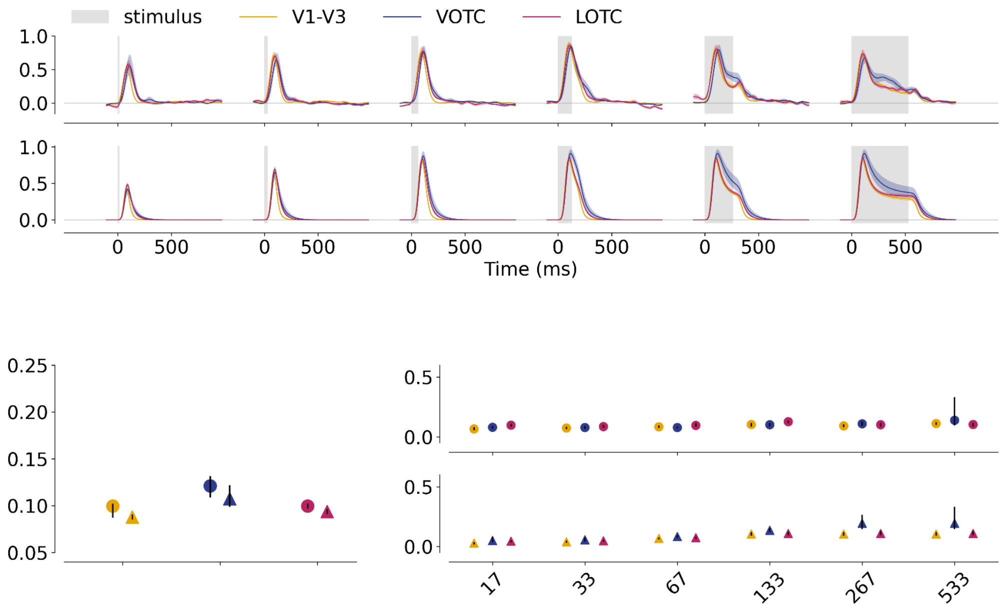

Additional Figure 1: Broadband responses across visual areas for single stimuli plotted separately for sub-p13 (panel **A**, number of electrodes: V1-V3, n = 3, VOTC, n = 4, LOTC n = 3) and sub-p14 (panel **B**, number of electrodes: V1-V3, n = 14, VOTC, n = 4, LOTC n = 6).

2. On page 15, the last paragraph, “from her on out” should be “from here on out”.

Action: Corrected text.

3. On page 25, line 3, “bbuildings” should be “buildings”.

Action: Corrected text.
