# Supplementary material for: Temporal dynamics of short-term neural adaptation across human visual cortex
Source: PLoS Comput Biol. 2024 May 30;20(5):e1012161. doi: 10.1371/journal.pcbi.1012161 (PMC11166327; doi:10.1371/journal.pcbi.1012161)
Supplement: Attachment — Submitted filename: comments_for_PCOMPBIOL_20240327.docx [file pcbi.1012161.s017.docx]

Comments for Brands et al., Temporal dynamics of short-term neural adaptation across human visual cortex (PCOMPBIOL-D-23-01549R1).

The revised manuscript has been carefully examined. I recognize the significant contributions this work presents. However, there remain a few nuanced areas that would benefit from additional refinement.

1. In the abstract, the author concludes that the study revealed temporal adaptation across the human visual hierarchy. However, upon review of Supplementary Table 3, it is evident that there is an imbalance distribution of electrodes among brain areas. This imbalance raises concerns regarding the sufficiency of using the term "visual hierarchy" as it is plausible that the unevenly distributed electrodes across brain areas may not adequately represent the entirety of the visual hierarchy. It is suggested that a more cautious interpretation could be considered to improve the validity of the findings.

2. On page 15, the last paragraph, “from her on out” should be “from here on out”.

3. On page 25, line 3, “bbuildings” should be “buildings”.
